# Structure-guided engineering of a mutation-tolerant inhibitor peptide against variable SARS-CoV-2 spikes

**DOI:** 10.1073/pnas.2413465122

**Published:** 2025-01-24

**Authors:** Shun Nakamura, Yukihiro Tanimura, Risa Nomura, Hiroshi Suzuki, Kouki Nishikawa, Akiko Kamegawa, Nobutaka Numoto, Atsushi Tanaka, Shigeru Kawabata, Shoichi Sakaguchi, Akino Emi, Youichi Suzuki, Yoshinori Fujiyoshi

**Affiliations:** ^a^Cellular and Structural Physiology Laboratory, Advanced Research Initiative, Institute of Integrated Research, Institute of Science Tokyo, Bunkyo-ku, Tokyo 113-8510, Japan; ^b^CeSPIA Inc., Taisei Otemachi, Chiyoda-ku, Tokyo 100-0004, Japan; ^c^International Center for Structural Biology, Research Institute for Interdisciplinary Science, Okayama University, Kita-ku, Okayama 700-8530, Japan; ^d^Division of Research Animal Laboratory and Translational Medicine, Research and Development Center, Osaka Medical and Pharmaceutical University, Takatsuki, Osaka 569-8686, Japan; ^e^Department of Pathology, Faculty of Medicine, Osaka Medical and Pharmaceutical University, Takatsuki, Osaka 569-8686, Japan; ^f^Department of Microbiology and Infection Control, Faculty of Medicine, Osaka Medical and Pharmaceutical University, Takatsuki, Osaka 569-8686, Japan

**Keywords:** SARS-CoV-2, spike, peptide engineering, structural biology

## Abstract

Pathogen mutations present an inevitable and challenging problem for therapeutics and the development of mutation-tolerant anti-infective drugs to strengthen global health and combat evolving pathogens is urgently needed. While spike proteins on viral surfaces are attractive targets for preventing viral entry, they mutate frequently, making it difficult to develop effective therapeutics. Here, we used a structure-guided strategy to engineer an inhibitor peptide against the SARS-CoV-2 spike, called CeSPIACE, with mutation-tolerant and potent binding ability against all variants to enhance affinity for the invariant architecture of the receptor-binding domain (RBD). High-resolution structures of the peptide complexed with mutant RBDs revealed a mechanism of mutation-tolerant inhibition. CeSPIACE bound major mutant RBDs with picomolar affinity and inhibited infection by SARS-CoV-2 variants in VeroE6/TMPRSS2 cells (IC_50_ 4 pM to 13 nM) and demonstrated potent in vivo efficacy by inhalation administration in hamsters. Mutagenesis analyses to address mutation risks confirmed tolerance against existing and/or potential future mutations of the RBD. Our strategy of engineering mutation-tolerant inhibitors may be applicable to other infectious diseases.

## Significance

During the recent COVID-19 pandemic, the pathogen SARS-CoV-2 continuously mutated to produce new strains, challenging the development of therapeutics. To develop a mutation-tolerant anti-infective drug, we focused on the fact that while pathogen proteins are variable, the core architecture responsible for the function indispensable for the pathogen life cycle never changes, making such invariant parts rational targets for anti-infectives. High-resolution structural information combined with mutagenesis assays enabled us to identify the invariant parts of the SARS-CoV-2 spike proteins and engineer an inhibitor peptide to enhance binding to the invariant parts. We demonstrated the efficacy of the peptide against various mutant spike proteins and authentic SARS-CoV-2 variants and confirmed its tolerance to existing and potential future mutations.

Infectious diseases are major threats to global health. Several coronaviruses and influenza viruses have caused large outbreaks in the 21st century: severe acute respiratory syndrome (SARS) in 2002–2003 (1, 2), novel influenzas in 2009 (3), Middle East respiratory syndrome in 2012 (4), and COVID-19 in 2019–2023 (5). Developing infection countermeasures is highly challenging due to pathogen mutations. Pathogens, especially viruses, mutate frequently to evade the immune system and render treatments ineffective. During the recent COVID-19 pandemic, the pathogen SARS coronavirus 2 (SARS-CoV-2) continuously mutated to produce new strains (6, 7), worsening global health and intensifying socioeconomic challenges (8). SARS-CoV-2 persists after the pandemic, posing the threat of aftereffects known as long COVID (9), and may continue to evolve into new variants. Newly developed mRNA vaccines, neutralizing antibodies, and oral drugs contributed to containing the pandemic (10–12), but their use was limited or revoked due to efficacy and safety issues, such as side effects, drug–drug interactions, or ineffectiveness resulting from mutations (13–18). In particular, mutations are complex, unpredictable, and difficult to address. Therefore, effective approaches are needed to develop long-term anti-infective drugs with mutation tolerance to combat ever-mutating pathogens. To address this need, we devised and implemented the strategy described below to design a mutation-tolerant inhibitor of SARS-CoV-2 infection.

**Fig. 1.**
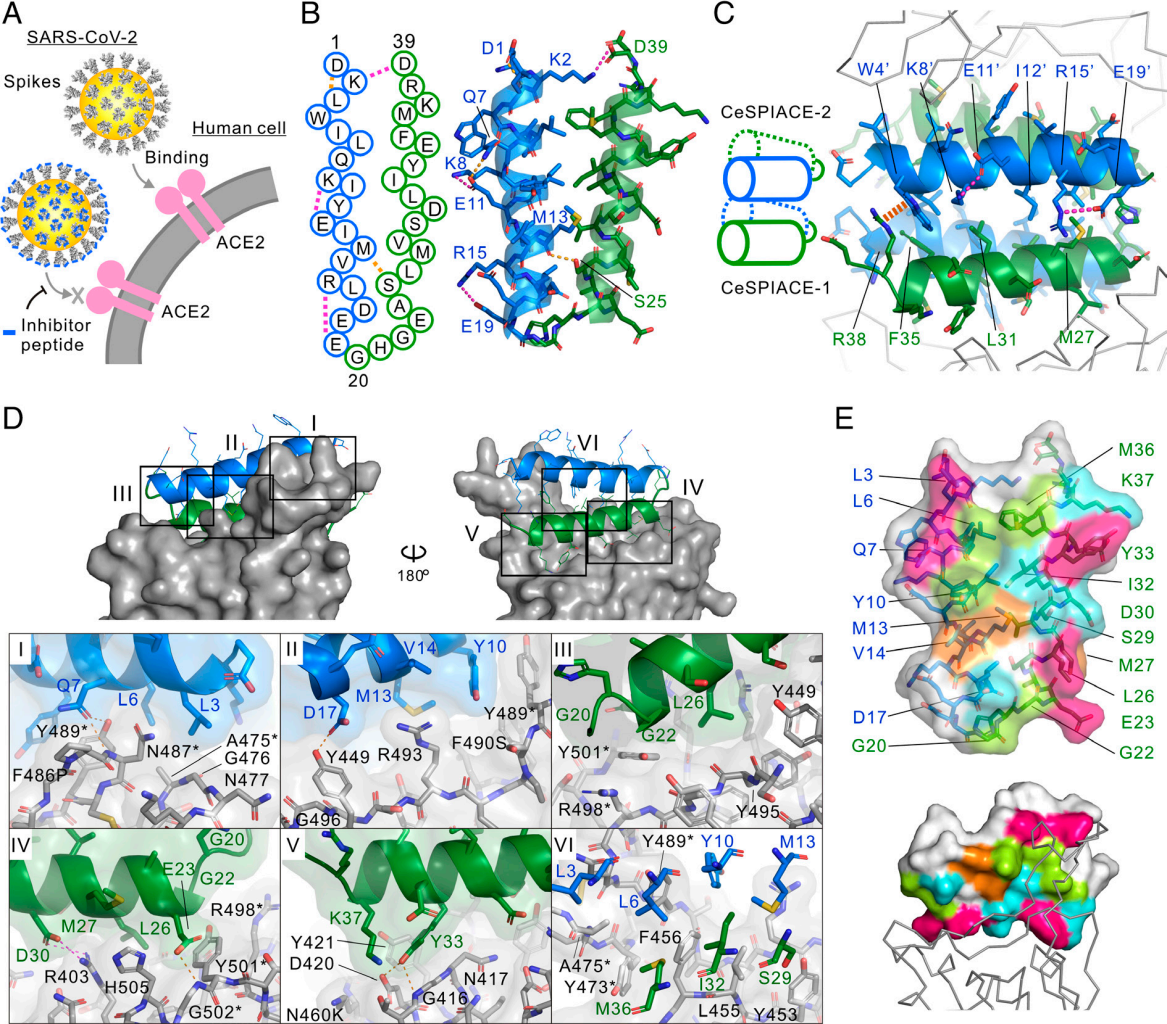
Structure-guided engineering of CeSPIACE. (*A*) Development strategy for therapeutics against COVID-19. Inhibitor peptides specifically bind to the SARS-CoV-2 spikes, competitively inhibiting RBD-ACE2 binding. (*B*) Amino acid sequence (*Left*) and helix bundle (*Right*) of CeSPIACE. The first helix consists of residues 1 to 19 in blue. The linker and second helix consist of residues 20 to 39 in green. Hydrogen bonds and salt bridges are indicated by orange and magenta dots, respectively. (*C*) Dimerization of CeSPIACE. The dimer is placed with one peptide at the *Bottom* and the other at the *Top* (schematic indication on the *Left*). The residue numbers of the *Upper* peptide are primed. A cation–pi interaction is shown as brown oval dots. Parts of the two RBDs are displayed as gray ribbon models above and below the CeSPIACE dimer (*Right*). (*D*) CeSPIACE-RBD binding structure. The XBB.1.5-type RBD is displayed as a gray surface model. Boxed areas with Roman numerals are enlarged below (I, III: *Top* view). Critical residues for ACE2 binding are marked with asterisks. (*E*) Binding surface of CeSPIACE. CeSPIACE is shown in the *Upper* model at the same angle as in (*B*), and in complex with the RBD in the *Lower* model. Surfaces binding to the main chain, to critical sites for ACE2 binding, and to other sites are shown in pink, greenish yellow, and cyan, respectively, and mutation-admissible surfaces are shown in orange.

Viral proteins are promising targets for therapeutic drugs in terms of efficacy and safety because therapeutics can act directly on the virus without interfering with human cell function. The spike protein of the SARS-CoV-2 binds to human angiotensin-converting enzyme 2 (ACE2) through the receptor-binding domain (RBD) and changes the conformation to fuse the viral and cell membranes, mediating viral entry (19–22). The spike RBD is one of the most attractive inhibitory targets because its inhibition prevents viral entry and does not require intracellular uptake (Fig. 1*A*). The spike protein is not only important for viral infection but also serves as a target for the immune system, resulting in frequent escape mutations. Many candidate treatments for spike protein-targeted inhibition, including antibodies or protein-based drugs, have been studied (23–27), but emerging variants rendered or would render them ineffective because they were not designed to recognize these mutations. For example, LCB1, a de novo*-*designed 56-mer miniprotein inhibitor, was meticulously designed to bind tightly to the surface of the wild-type (WT) RBD (23) but its efficacy was substantially attenuated by mutations in existing SARS-CoV-2 variants, including the Omicron strain (28, 29). The relatively large interface of protein–protein interactions (PPIs) provides high affinity for flat surfaces like RBDs but may increase the opportunity for escape mutations. On the other hand, increasing universality to various interfaces by decreasing the contact area may lead to reduced affinity and specificity. Here, we used a structure-guided strategy to engineer an inhibitor peptide with mutation-tolerant and potent binding ability against all SARS-CoV-2 variants. To solve the conflict between universality and affinity, we focused on the fact that variable pathogen proteins do not alter the essential structure required for functions indispensable to the viral life cycle; for the SARS-CoV-2 spike proteins, this indispensable function is binding to ACE2. We identified the invariant architecture shared by variant RBDs via structural analysis and structure-guided mutagenesis. Modifying the peptide to enhance binding to the invariant parts increased both the universality and affinity of the protein. We named the peptide CeSPIACE (COVID-19 eliminative Short-Peptide Inhibiting ACE2 binding).

## Results

### Structure-Guided Peptide Engineering.

We engineered mutation-tolerant CeSPIACE based on high-resolution structural information in combination with binding assays. The engineering started by referencing the RBD-binding part of LCB1 as a binding moiety for the ACE2-binding site of the RBD despite its vulnerability to mutations. We identified a 39-mer peptide region that was easily chemically synthesized and modified it to improve structural stability and mutation tolerance. For engineering, we determined approximately 2 Å-resolution crystal structures of CeSPIACE or its intermediate-stage peptides complexed with the RBDs of several strains, including WT, Alpha, Delta, BA.2, BA.5, or XBB.1.5 (*SI Appendix*, Fig. S1 and Table S1). The high-resolution structures identified the roles of the peptide residues and provided information on which residues to modify and how to modify them (*SI Appendix*, Table S2). CeSPIACE formed a two-helix bundle (Fig. 1*B*) and homodimerized to form a four-helix bundle with the RBD-binding site facing outward (Fig. 1*C* and *SI Appendix*, Fig. S1 *C*and *D*); a similar dimer was reported by Khatri et al (26). We introduced notable interactions to form a rigid helix bundle; a salt bridge between R15-E19 to stabilize the helix, K2-D39 interactions at the edge to strengthen the helix bundle, and a cation–pi interaction between W4-R38 to maximize the dimer interface (Fig. 1 *B* and *C* and *SI Appendix*, Fig. S1*E*). Surface plasmon resonance (SPR) analyses (30) showed that these interactions increased the RBD affinity despite not directly interacting with the RBD (*SI Appendix*, Fig. S2 *A* and *C*), presumably due to stabilizing the ready-to-bind structure.

RBD mutations have occurred at more than 30 residues among the SARS-CoV-2 variants that have been under World Health Organization (WHO) surveillance, including variants of concern (VOCs), variants of interest (VOIs), and variants under monitoring (VUMs) (*SI Appendix*, Fig. S3). The binding interface of the RBD with CeSPIACE is largely shared with that to ACE2 (*SI Appendix*, Fig. S6*A*), so mutations resulting in immune evasion outside the ACE2-binding site do not affect the affinity to CeSPIACE. Binding only to the ACE2-binding site, however, is insufficient to address mutations. Even residues on the ACE2-binding site are potential options for mutation if the immune evasion advantage outweighs the disadvantage of the reduced affinity, such as K417N, F486V, and F486S (*SI Appendix*, Fig. S3). Therefore, we enhanced CeSPIACE to recognize the invariant architecture of the RBD regardless of mutations. First, the main-chain structure of the ACE2-binding site is invariant to form the backbone of the binding site, though amino acid substitutions alter the side chain. Superimposition of the crystal structures confirmed that the RBD main chain of the binding interface was perfectly aligned (*SI Appendix*, Fig. S5*A*), and the main-chain structures on RBD surfaces were invariant among various mutants (*SI Appendix*, Fig. S5*B*). L3, Q7, E23, L26, and Y33 of CeSPIACE recognize the main-chain structure of RBD around A475-G476, N487, Y501-G502, Y495, and G416-N417, respectively, fitting into the groove between side chains (Fig. 1*D* and *SI Appendix*, Fig. S5*C*). RBD residues essential for ACE2 binding are also good targets as invariant parts. ACE2 binding is indispensable for the SARS-CoV-2 life cycle, so mutations losing ACE2-binding ability do not occur. Structure-guided mutagenesis of the RBD revealed critical residues for ACE2 binding, which were evaluated by peak-shift assays between ACE2 and single mutants of the BA2-type RBD in fluorescence size-exclusion chromatography (FSEC) (*SI Appendix*, Fig. S6 *B* and *C*). On the CeSPIACE-binding site, Y489 and G502 were extremely critical, as even a small change, Y489F or G502A, prevented ACE2 binding. Y473, A475, N487, R498, and Y501 were also important for ACE2 binding, as they could not tolerate substitutions with residues of different properties and significantly reduced the binding ability. Crystal structures of ACE2 confirmed the importance of these residues tightly bound to the RBD (*SI Appendix*, Fig. S6*D*). Mutational scanning using yeast-displayed WT RBD supports these results (31). CeSPIACE was designed to interact with Y473, A475, N487, and Y489 via L3, L6, Q7, Y10, and M36; with R498 via G20; and with Y501 and G502 via G22, E23, and L26 (Fig. 1*D* and *SI Appendix*, Fig. S4). Additionally, we engineered the binding moieties to avoid steric hindrance and electrical repulsion with mutations. Y501 of the RBD is an essential residue after the Omicron strain, but it was N501 in the WT and Delta strains (*SI Appendix*, Fig. S3*A*). The binding surface composing of G22 and L26 contributed to binding to both tyrosine and asparagine, whereas the previous A22 and M26 reduced the binding ability to Y501 (*SI Appendix*, Fig. S2 *B* and S4 *C*). M13 and V14 form a neutral and optimally sized pocket to accommodate both Q493 and cationic R493, which exist among Omicron subvariants (*SI Appendix*, Figs. S3 *C* and S4 *B*). M27 improved accessibility to bulky residues Y505 and H505 by extending the side chain toward the dimer interface (*SI Appendix*, Fig. S4*D*). The mutation-admissible interfaces may cause varying affinity to some extent depending on the mutation type but can avoid critical hindrance, and the binding on the invariant regions complemented the affinity. We comprehensively designed the binding surface to potently bind to various mutant RBDs (Fig. 1*E*).

### Binding and Inhibitory Activity.

**Fig. 2.**
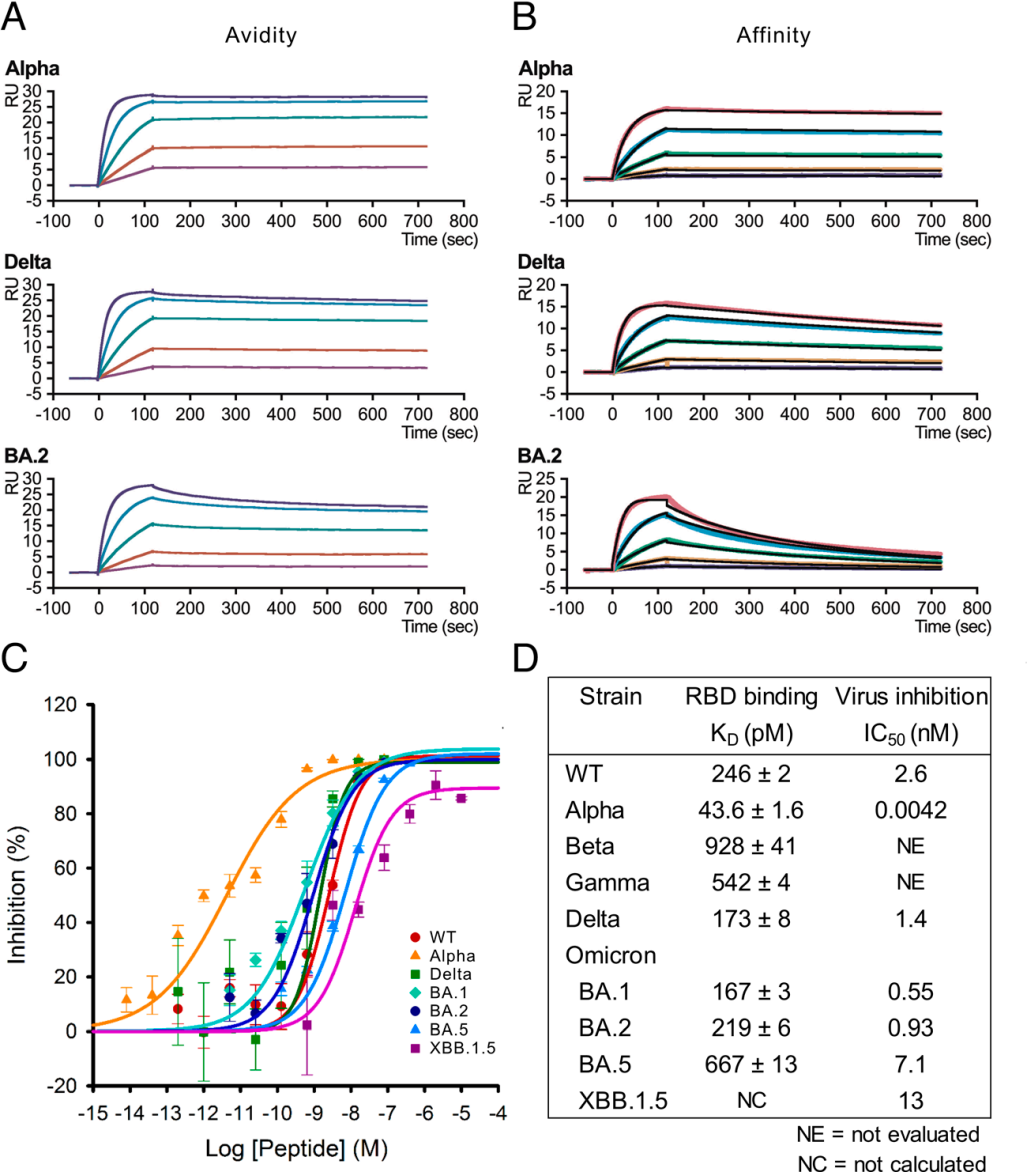
Binding and inhibitory activities of CeSPIACE. (*A*) SPR measurements for qualitative analyses against variant RBDs. Datasets of three representative variants are shown, and other variants are shown in *SI Appendix*, Fig. S7. CeSPIACE concentrations were prepared stepwise; 5, 10, 20, 40, and 80 nM. (*B*) SPR measurements for quantitative analyses. The best fit of the data to a 1:1 binding model is shown by the black line. (*C*) Inhibition curves in antiviral assay using VeroE6/TMPRSS2 cells. Fitting curves were calculated from the data from experiments repeated five times for WT, Alpha, and Delta; three times for BA.1, BA.2, and XBB.1.5; and nine times for BA.5 (mean ± SE). (*D*) K_D_ and IC_50_ values. Kinetic parameters were calculated from the data in (*B*) and *SI Appendix*, Fig. S7 *B* and *D*, from three independent experiments. The K_D_ of the XBB.1.5 strain could not be calculated due to measurement issues. The IC_50_ was calculated from the data in (*C*).

We evaluated the binding potency of CeSPIACE to RBDs using SPR. Qualitative binding profiles were measured using a sensor chip with immobilized RBDs. SPR analyses showed that CeSPIACE bound rapidly and rarely dissociated against RBDs of various strains, including WT, Alpha, Beta, Gamma, Delta, Omicron BA.1, BA.2, BA.5, and XBB.1.5 (Fig. 2*A* and *SI Appendix*, Fig. S7*A*). The nondissociative property indicated that CeSPIACE would likely retain its ability to prevent viral infection for a long time. Dimeric CeSPIACE has divalent binding sites, similar to antibodies, so these binding profiles reflect the functional avidity of the accumulated affinities of all the binding sites (32). The divalent binding sites bridged two RBDs on the sensor chip, making it difficult to measure affinity on the basis of the strength of a single binding site (*SI Appendix*, Fig. S7*C*). To calculate the affinity of CeSPIACE-RBD binding, we performed SPR analysis using a sensor chip with immobilized tandem-linked CeSPIACE, which provided a 1:1 binding model for evaluating the affinity (*SI Appendix*, Fig. S7*C*). SPR analyses revealed that CeSPIACE bound to major mutant RBDs with picomolar affinity, ranging from 44 pM to 928 pM (Fig. 2 *B* and *D* and *SI Appendix*, Fig. S7*B*). CeSPIACE exhibited high affinity to all mutant RBDs, but the kinetics differed depending on the mutant, with the binding rate being fastest for the Omicron-type RBD and the dissociation rate being slowest for the Alpha-type RBD (*SI Appendix*, Fig. S7*D*). We confirmed in the crystal structures that the differences in kinetics were due to some typical mutations. Y501 binds more tightly on G22 and L26 than N501 (*SI Appendix*, Fig. S4*C*), which contributes to the nondissociation properties of the Alpha strain. K417 interacts with D30, but N417 does not (*SI Appendix*, Fig. S4*E*), which increases the dissociation rate of strains such as Beta and Omicron. The longer side chain of R493 contacts the binding surface comprising M13 and V14 more extensively than Q493 (*SI Appendix*, Fig. S4*B*), resulting in retention of the binding state.

We investigated the inhibitory activity of CeSPIACE against authentic SARS-CoV-2 variants. Plaque reduction assays using VeroE6/TMPRSS2 cells (33) showed that CeSPIACE potently neutralized the variants, including Alpha, Delta, BA.1, BA.2, BA.5, and XBB.1.5 (Fig. 2*C*). The inhibitory activity of CeSPIACE against 100 plaque-forming units (PFU) per well of SARS-CoV-2 variants was in the nanomolar or lower range against all variants, with the half-maximal inhibitory concentration (IC_50_) ranging from 4 pM to 13 nM (Fig. 2*D*). The inhibitory activity against viruses correlated well with the equilibrium dissociation constant (K_D_) against RBDs measured by SPR, indicating that the high affinity to the RBD was effective for preventing viral infection. Although a virus has dozens of spike trimers to block, low concentrations of CeSPIACE effectively inhibited viral entry. The potent inhibition against the various strains demonstrated that our peptide engineering strategy worked well against authentic SARS-CoV-2.

### Therapeutic Efficacy.

**Fig. 3.**
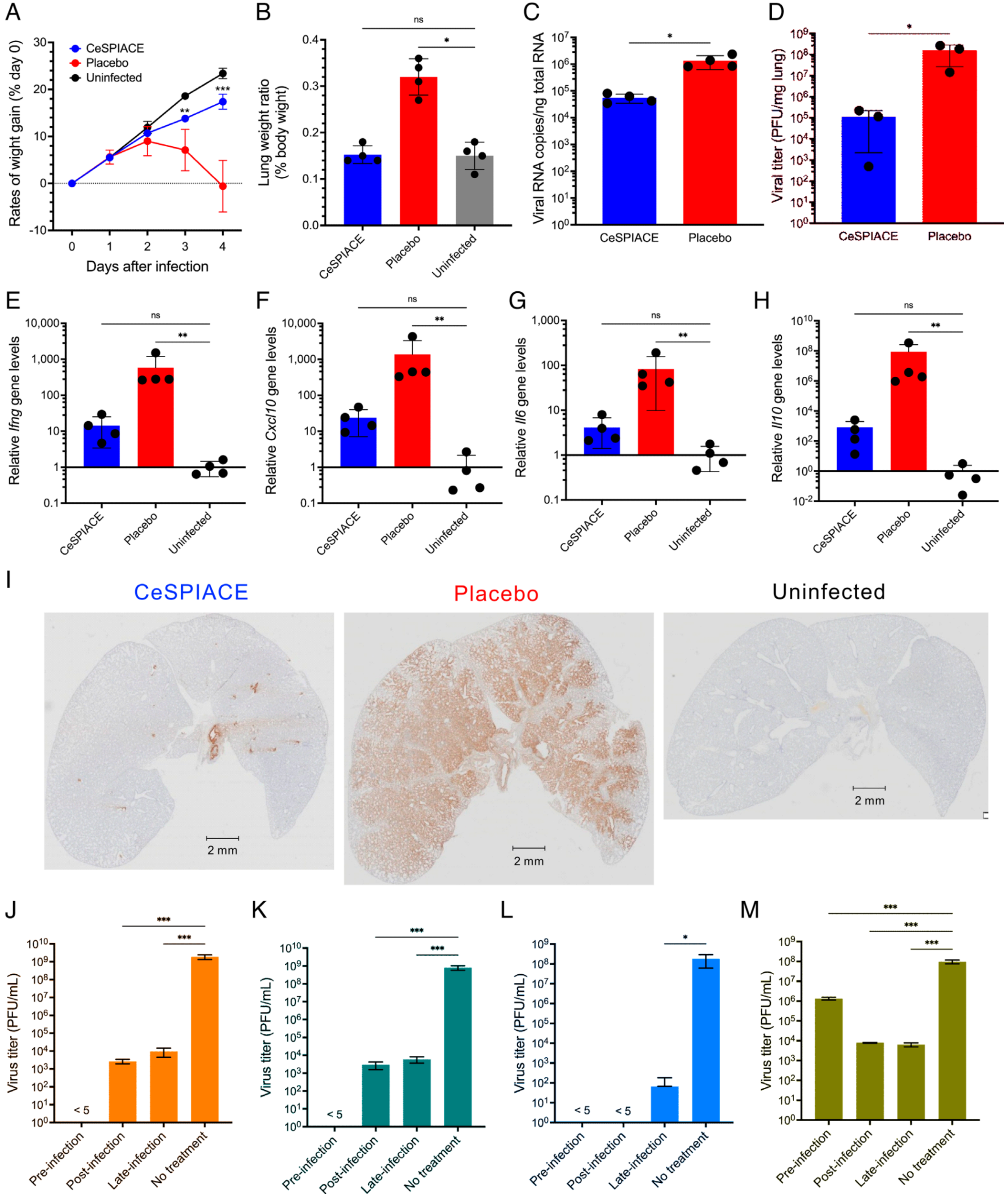
Therapeutic efficacies of CeSPIACE. (*A*) Body weight change of hamsters after inoculation (mean ± SD, n = 4). (*B*) Lung weight to body weight ratio of hamsters at 4 d postinfection. (*C*) Viral load in the lungs of hamsters at 4 d postinfection. (*D*) Viral titers of lung homogenates at 4 d postinfection. CeSPIACE was administered closer to the airway in this experiment, whereas in the other experiments, administration was into the oral pharynx. (*E–H*) Cytokine expression of IFN-gamma (*E*), CXCL10 (*F*), IL-6 (*G*), and IL-10 (*H*). (*I*) Immunostaining images of lung sections of hamsters at 4 d postinfection, stained by anti-SARS-CoV-2 nucleocapsid antibody. (*J–M*) Viral titers of cell culture media of Calu-3 cells infected by WT (*J*), Alpha (*K*), Delta (*L*), and Omicron BA.5 (*M*) viruses (mean ± SD). Viral titer undetectable with plaque-forming assay is indicated as < 5. Statistical significance was determined by two-way ANOVA (*A*), Kruskal–Wallis (*B* and *E*–*H*), Mann–Whitney (*C* and *D*), and one-way ANOVA (*J*–*M*) tests. *P* values below 0.05 (*P* < 0.05, *; *P* < 0.01, **; *P* < 0.001, ***) were considered significant. ns, not significant.

We confirmed the therapeutic efficacy of CeSPIACE by inhalation administration in a Syrian hamster model (34). SARS-CoV-2 infection begins in the nasal cavity and spreads to the respiratory tract (35), so we are developing CeSPIACE as an inhalant to directly prevent viral infection. We intranasally inoculated Syrian hamsters with 5,000 PFU of SARS-CoV-2 Delta variant, the most virulent strain, and then administered 0.5 µg (100 pmol) of CeSPIACE using a bronchial spray to the oral pharynx 1 h after the infection and once daily for 3 more days (*SI Appendix*, Fig. S8*A*). CeSPIACE treatment prevented weight loss and reduced the viral burden and lung damage. The hamsters administered CeSPIACE gained body weight similarly to noninfected hamsters, whereas the placebo-administered hamsters gained significantly less weight or lost weight (Fig. 3*A* and *SI Appendix*, Fig. S8*B*). Although lung damage by infection caused lung edema and consequently increased lung weight, the lung weight to body weight ratio in the CeSPIACE-administered hamsters was similar to that of noninfected hamsters (Fig. 3*B* and *SI Appendix*, Fig. S8*C*). The viral RNA levels were lower in the lungs of CeSPIACE-administered hamsters compared with those of the placebo-administered hamsters (Fig. 3*C*). The viral titers were reduced by three orders of magnitude through administering closer to the airway (Fig. 3*D*). Pathologic anatomic analysis showed that CeSPIACE-treated lungs were essentially similar to uninfected lungs. Lung sections from the placebo-administered group, but not in the CeSPIACE-administered and uninfected groups, showed inflammation characterized by airspace consolidation and immune cell infiltration (*SI Appendix*, Fig. S8*D*). The total area of pathologic lesions in lungs treated with CeSPIACE was significantly smaller than that in lungs treated with placebo (*SI Appendix*, Fig. S8*E*). Inflammatory cytokine RNA levels were significantly lower in the CeSPIACE-treated lungs than in the placebo-treated lungs, suggesting that CeSPIACE treatment prevents viral infection and inflammation in the lung (Fig. 3 *E*–*H*). Immunostaining of lung sections with anti-SARS-CoV-2 nucleocapsid antibody revealed that CeSPIACE protected lungs against viral infection (Fig. 3*I* and *SI Appendix*, Fig. S8*F*). These results demonstrated the potent in vivo efficacy of CeSPIACE even with low-dose inhalation administration.

We performed a time-of-addition (ToA) assay using human lung-derived Calu-3 cells to investigate the steps in the viral life cycle on which CeSPIACE exerts its therapeutic effects. We evaluated the antiviral activity of CeSPIACE against WT, Alpha, Delta, and Omicron BA.5 viruses, adding CeSPIACE at different times: premixed with the virus (pretreatment), at 2 h after inoculation (postinfection), or 18 h after inoculation (late-infection; Fig. 3 *J–M* and *SI Appendix*, Fig. S9). The ToA assay showed potent antiviral activities against all variants at all steps. CeSPIACE inhibited not only initial viral entry but also subsequent rounds of reinfection. The ToA assay results indicated that CeSPIACE is efficacious for inhibiting infection of human cells from prophylaxis to postinfection treatment.

### Cryo-EM Structures of CeSPIACE and Spike Complex.

**Fig. 4.**
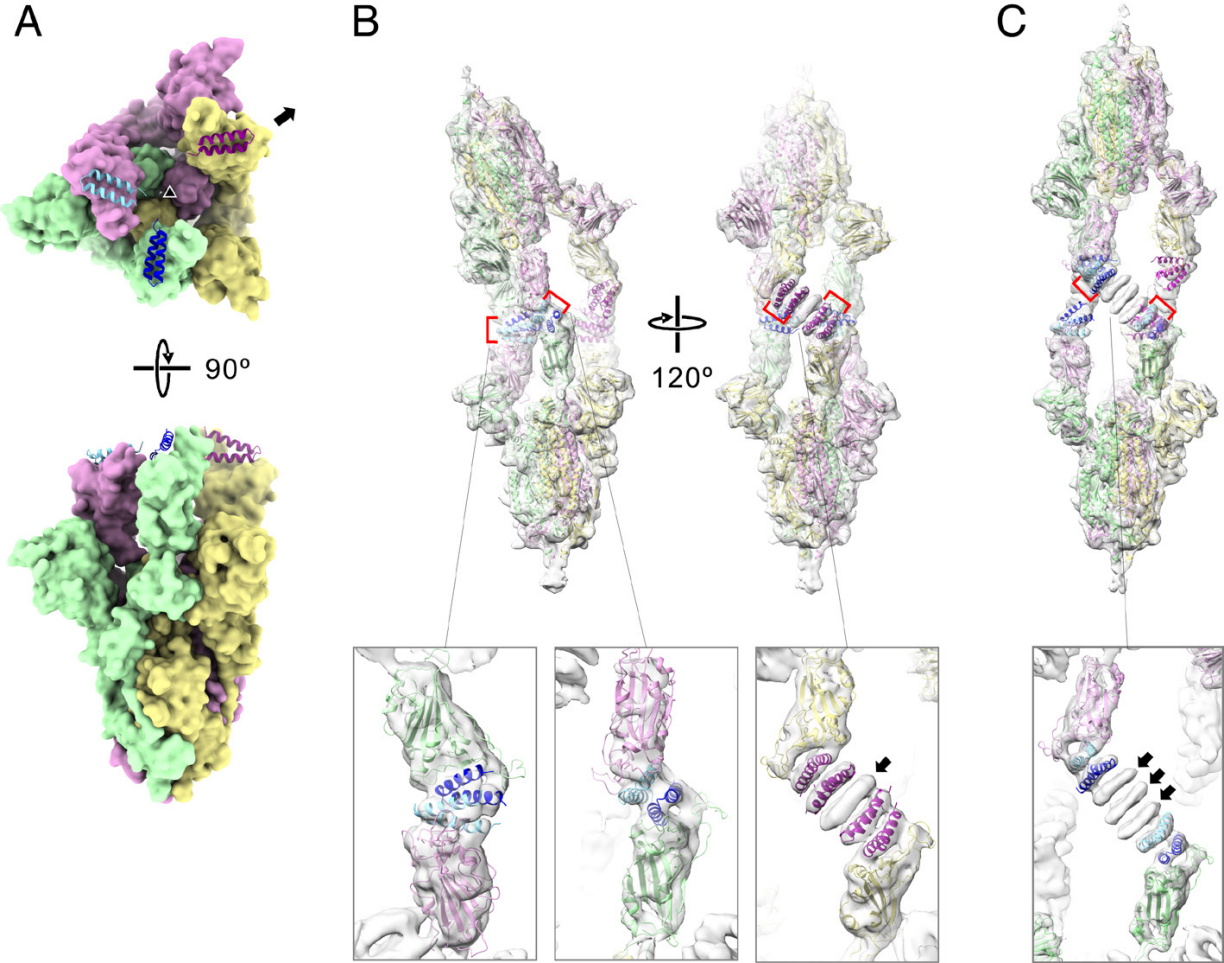
Cryo-EM structures of CeSPIACE-spike ectodomain complex. (*A*) Structure of CeSPIACE-bound spike of BA.2 and the CeSPIACE monomers, shown in surface and ribbon representation, respectively. A triangle indicates the pseudo-three-fold symmetry axis, and an arrow indicates the displacement of one RBD from the symmetry by binding the CeSPIACE dimers. (*B*) Side view density maps of CeSPIACE dimer binding to spikes of BA.2 are shown from two different directions. Enlarged maps are also shown below. Each of two pairs of RBDs was strongly connected by one CeSPIACE dimer and two spikes arranged in a bent manner. As a result, another pair of RBDs was connected by two CeSPIACE dimers (magenta) and monomeric CeSPIACE (arrow, 5 peptides in total). (*C*) Side view density maps of CeSPIACE dimer binding spikes of BA.5 (class II) are shown. The enlarged map of the CeSPIACE binding region is shown below. In the case of CeSPIACE dimer binding spikes, the two-spike configuration is rather straight. Each pair of RBDs was connected by two CeSPIACE dimers (light and dark blue) and three monomeric CeSPIACE (arrows, 7 peptides in total).

We determined the cryo-EM structures of CeSPIACE complexed with spike ectodomain trimers whose RBDs were BA.2 or BA.5-type (*SI Appendix*, Fig. S10 and Table S3). The EM map showed that CeSPIACE binds to each RBD of the trimer, forcing three RBDs into an open conformation (Fig. 4*A*), although in the prefusion state, the trimer is predominantly in a single RBD open conformation (20, 21). Two spike trimers formed a head-to-head complex mediated by CeSPIACE involving several docking modes. A typical docking complex occurred when the CeSPIACE-RBD binding restricted the orientation of a head-to-head complex, thereby twisting the overall architecture and distorting the trimer symmetry (Fig. 4*A*). Two spike trimers docked via CeSPIACE dimers at two of the sites but via a stack of multiple peptides at the third site (Fig. 4*B* and *SI Appendix*, Fig. S10). Conformational restriction induced by dimer-mediated binding at two sites created a wider space between the third pair of the opposing RBDs, and two peptide dimers and an additional peptide filled the resulting space (Fig. 4*B*, arrow). Another docking complex was observed in the BA.5-type spike, in which two spike trimers docked via seven peptides at three sites (Fig. 4*C* and *SI Appendix*, Fig. S10 *H*, *M*, and *N*). Most particles consisted of the docking complex linked at all three sites (*SI Appendix*, Fig. S10 *A*, *H*), suggesting that triple-linked complexes are more stable than single- or double-linked complexes, which have wobbly sites. The crystal structures and peak retention time in gel filtration indicated that CeSPIACE certainly dimerized in physiologic solutions, but the cryo-EM structures suggested that CeSPIACE has stacking properties that allow it to dock two spikes. The stacking number of CeSPIACE varied, but relatively numerous peptides may render sufficient flexibility to dock two spike trimers distorted by the potent binding with CeSPIACE. Head-to-head complexes similar to the cryo-EM structures would form on the viral envelope, implying that CeSPIACE can bridge separate spikes to aggregate viral particles. Based on a pseudovirus neutralization assay and dynamic light scattering analysis, it was reported that another peptide linked spikes to aggregate viruses, thereby enhancing inhibitory activity through the aggregation effect (26). Antibodies like IgM are also known to form agglutinating complexes, inducing pathogen aggregation in the immune system (32). The spike–spike linking ability of CeSPIACE may contribute to enhancing its inhibitory activity and facilitating pathogen elimination.

### Tolerance Against Existing and/or Possible Future Mutations.

**Fig. 5.**
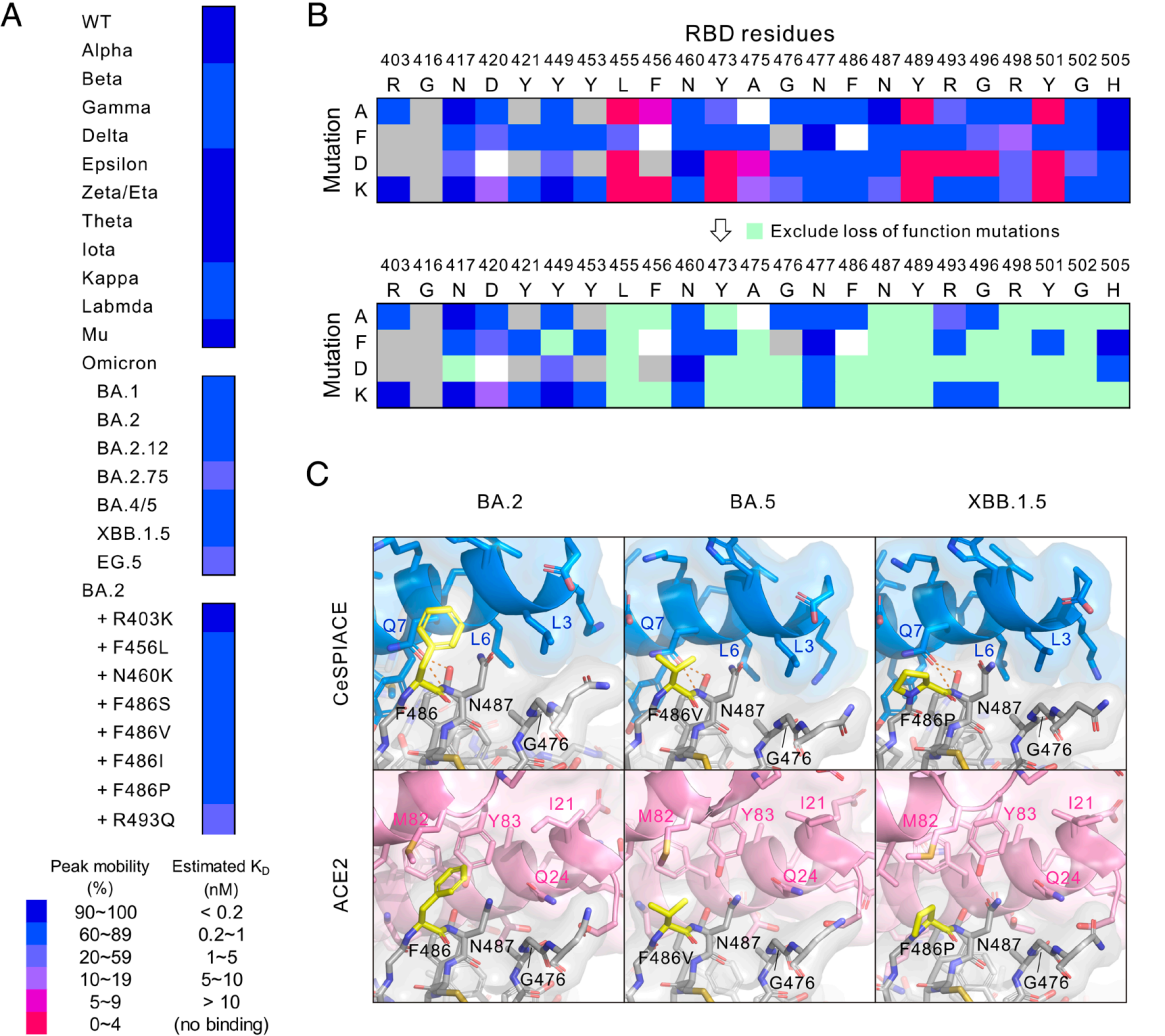
Tolerance against existing and/or possible future mutations. (*A*) CeSPIACE affinity for existing mutants. The affinities were measured by the peak-shift assay and colored according to the peak mobility correlated with K_D_ of SPR as shown in the legend. (*B*) CeSPIACE affinity heatmap of possible RBD mutations. The *Top* panel includes all results, and the *Bottom* panel excludes loss-of-function mutations. Unexpressed mutants are colored gray, indicating that folding was affected. (*C*) Binding structures around the F486 mutations. CeSPIACE, ACE2, and RBD are shown in blue, pink, and gray, respectively. F486 or F486 mutations are highlighted in yellow. The PDB codes of ACE2 structures are as follows. ACE2-RBD BA2: 7XB0, ACE2-RBD BA.5: 7XWA, ACE2-RBD XBB.1.5: 8SPI.

We examined the mutation tolerance of CeSPIACE against variants that have ever been under WHO surveillance, including VOCs, VOIs, and VUMs (*SI Appendix*, Fig. S3). To efficiently assess the affinity against numerous mutant RBDs, we performed a peak-shift assay in FSEC, where peak mobility by binding was correlated with the K_D_ of SPR (*SI Appendix*, Fig. S11 *A* and *B*). The peak-shift assay showed that CeSPIACE potently bound to all WHO-labeled variants (Alpha through Omicron) and major Omicron subvariants, including BA.1, BA.2, BA.2.12, BA.2.75, BA.4/5, XBB.1.5, and EG.5, with nanomolar or subnanomolar affinity (Fig. 5*A*). Mutation mapping on the structure showed that most mutations of the Omicron subvariants were outside the CeSPIACE-binding site, but R403K, F456L, N460K, F486S/V/I/P, and R493Q were on the binding site (*SI Appendix*, Fig. S3). FSEC analyses showed that CeSPIACE can also potently bind to these binding-site mutations (Fig. 5*A*). These results showed that CeSPIACE can inhibit all existing variants with high affinity.

Unknown spike mutations may affect CeSPIACE affinity in the future. We, therefore, evaluated the effects of representative mutations on all 23 residues of the CeSPIACE-binding site using the peak-shift assay. Escape mutation tends to alter the size or charge of its side chains, so we substituted an RBD residue of the BA.2 strain, which is the origin of current variants, to a short, bulky, anionic, or cationic residue: alanine, phenylalanine, aspartic acid, or lysine, respectively. Mutations with significantly reduced ACE2 binding ability, such as the Y489 mutations (*SI Appendix*, Fig. S6*C*), were excluded from the evaluation as loss-of-function mutations. The peak-shift assay showed that most mutants maintained or enhanced the CeSPIACE affinity even though the ACE2 affinity was reduced in some cases (Fig. 5*B*). A few mutants, such as D420F and D420K, reduced the CeSPIACE affinity but retained nanomolar affinity, which is acceptable for therapeutics. We performed additional analyses on D420, N460, and F486 to address their probable risks. D420 and N460 contact CeSPIACE but not ACE2 (*SI Appendix*, Fig. S6*A*), so mutations may easily occur. F486 recently emerged as a mutational hotspot (*SI Appendix*, Fig. S3*C*) and may mutate to another residue. Detailed mutagenesis analyses using peak-shift assays confirmed that N460 or F486 mutations had little effect on CeSPIACE affinity, and while some D420 mutations slightly decreased the affinity, the affinities were still sufficiently high (*SI Appendix*, Fig. S11*D*). The F486 mutations tended to reduce the ACE2 affinity but did not affect the CeSPIACE affinity because CeSPIACE recognizes the RBD main chain through L3 and Q7 whereas ACE2 primarily interacts with the F486 side chain (Fig. 5*C* and *SI Appendix*, Fig. S11 *C* and *D*). These results, therefore, showed that CeSPIACE has mutation tolerance against possible future variants.

## Discussion

Pathogen mutation is an inevitable problem in infection countermeasures, so efficacy against all variants, not limited variants, is critical for developing long-term applicable therapeutics. Although drug–target binding is strengthened by recognizing the specific shape or charge of the target, a specificity that is too high is more susceptible to disruption by mutation. One of our intermediate peptides, Ce149, potently inhibited mutant RBDs up to the BA.2 strain by enhancing a hydrophobic interaction with F486, but its efficacy was attenuated by the F486V mutation of the BA.5 strain (*SI Appendix*, Fig. S2*D*) due to the high specificity for the F486 side chain, making it vulnerable to mutation. To solve the conflict between specificity and universality in antiviral treatments, we engineered CeSPIACE to recognize the invariant architecture of the RBD critical for its function, thereby achieving mutation-tolerant affinity. For structure-guided engineering, it is important to understand the binding mechanism through basic research and to utilize structural information. Drug design has been challenging using conventional high-throughput screening in combinatorial libraries of low-molecular weight compounds or cyclic peptides (36), which can lead to off-target binding. Remarkable advances in structural biology make it possible to rationally engineer therapeutic peptides or proteins. High-resolution crystal and cryo-EM structures allow us to control PPI at the atomic level to target specific sites. Structure prediction technology using AI programs has rapidly progressed in recent years and facilitates the design of de novo peptides (37, 38). Incorporating our strategy of targeting invariant regions may facilitate effective computational drug discovery of anti-infective drugs.

Peptides have great potential as therapeutics. They can have high specificity and affinity like antibodies and are capable of structure-guided PPI control, resulting in fewer side effects and no drug–drug interactions. Their relatively small molecular weight allows for chemical synthesis, contributes to less immunogenicity, and enables more flexible and versatile dosing options. In addition to the efficacy of CeSPIACE discussed in this paper, its high safety was confirmed by a dose range-finding study, and we obtained prospective preclinical results in collaboration with a pharmaceutical company. CeSPIACE consists of 39 natural amino acids and can be mass-produced through chemosynthesis; its stability has been confirmed in powder and solution form. Our preclinical experiments demonstrated that CeSPIACE is efficacious through inhalation using a nebulizer or dry powder. Biologics, such as antibodies, are increasingly being approved for clinical use and are actively developed for application in drug delivery systems like antibody-drug conjugates (39). Like antibodies, peptides have high binding ability through PPIs and can be used as alternatives in approaches similar to those used with antibodies. CeSPIACE has potential applicability to drug delivery systems targeting SARS-CoV-2 and virus detection systems like antigen tests.

All pathogen proteins, like the SARS-CoV-2 spike, have invariant structures critical for their functions, which can be good targets for mutation-tolerant drugs as in our peptide engineering; the essential structure of hemagglutinin binding to sialic acid in the influenza virus or gp120 binding to CD4 in the HIV are other potential targets. Unknown infectious diseases will continue to emerge. Our strategy of engineering mutation-tolerant inhibitors can apply to the development of therapeutics against other existing infections or future pandemics.

## Materials and Methods

### Peptide Preparation.

Peptides were chemically synthesized using Fmoc-based chemistry and purified using reversed-phase high-performance liquid chromatography by Eurofins or Cosmo Bio Co. The peptide powders were dissolved in buffer containing 20 mM HEPES-NaCl (pH 8.0) and 150 mM NaCl. The peptides for animal experiments were dissolved in 50 mM HEPES-NaCl (pH 8.0). The peptide solution was filtered using Ultrafree MC HV centrifugal filters (Merck Millipore). The peptide concentration was measured by absorbance at 280 nm.

### Protein Expression and Purification of RBD.

The SARS-CoV-2 spike RBD (residues Arg319-Phe541 for binding assays in SPR and FSEC or Thr333-Gly526 for crystallization) with an N-terminal µ-phosphatase signal peptide and a C-terminal 10xHis tag and Avi tag (40) linked through a tobacco etch virus (TEV) protease recognition sequence was subcloned into a pFastBac1 plasmid modified based on a previous report for the BacMam expression system (41). Mutations were introduced to the RBD, as shown in *SI Appendix*, Fig. S3, but S375F of the Omicron strain was not introduced due to expression challenges. The recombinant baculovirus was generated according to the manufacturer’s instructions for Bac-to-Bac (Life Technologies) and transiently transfected into Expi293F cells in Expi293 expression medium (Gibco) with penicillin-streptomycin-amphotericin B (Fujifilm Wako) and 5 mM sodium butyrate for 72 h at 37 °C. The His-tagged protein was harvested from the cell culture medium, purified with TALON cobalt affinity resin (Clontech), and further purified by size-exclusion chromatography (SEC) using a Superdex200 Increase 10/300 column (Cytiva) in buffer containing 5 mM Tris-HCl (pH 8.0) and 50 mM NaCl for binding assays. The peak fractions were collected and concentrated to 3 mg mL^−1^ using an Amicon Ultra 4 MWCO 10-kDa filter (Merck Millipore).

For crystallization, the protein was prepared after His-tag affinity purification as follows. The buffer was exchanged with HEPES-buffered saline (HBS, 20 mM HEPES-NaCl [pH 7.0], and 150 mM NaCl) using a PD-10 desalting column (Cytiva). The eluate was incubated with TEV protease and PNGase F (New England Biolabs) overnight at 20 °C, and the 10xHis-Avi tag was removed by passing through TALON cobalt affinity resin. The RBD was mixed for 30 min with CeSPIACE or other peptides at a molar ratio of 1:1. The RBD/peptide was purified by SEC using a Superdex200 Increase 10/300 column (Cytiva) in HBS. The peak fractions were collected and concentrated to 20 mg mL^−1^ using an Amicon Ultra 4 MWCO 30-kDa filter (Merck Millipore).

### Protein Expression and Purification of the Spike Ectodomain.

The SARS-CoV-2 spike ectodomain (residues Ser13-Gln1208 with six proline substitutions (42) at residues 817, 892, 899, 942, 986, and 987 and GSAS substitution at residues 682-685) with an N-terminal µ-phosphatase signal peptide and a C-terminal foldon trimerization motif, 8xHis, and Twin-Strep tag was subcloned into the pFastBac1 plasmid described above. The plasmid was transiently transfected into Expi293F cells using polyethylenimines in Expi293 expression medium (Gibco) with penicillin-streptomycin-amphotericin B (Fujifilm Wako) and 5 mM sodium butyrate at 37 °C for 72 h. The ectodomain was harvested from the cell culture medium and purified with Strep Tactin resin (IBA). The ectodomain was mixed for 30 min with CeSPIACE at a molar ratio of 1:4. The ectodomain/peptide was purified by SEC using a Superose6 Increase 10/300 column (Cytiva) in buffer containing 2 mM Tris-HCl (pH 8.0), 200 mM NaCl, and 0.02% NaN_3_. The peak fractions were collected and concentrated to 1 mg mL^−1^ using a Vivaspin Turbo 4 MWCO 50-kDa filter (Sartorius).

### Protein Expression and Purification of ACE2.

The human ACE2 extracellular domain (residues Ser19-Asp615) with a C-terminal enhanced green fluorescent protein-10xHis-Flag tag linked through the TEV protease recognition sequence was subcloned into the pFastBac1 plasmid. The recombinant baculovirus was generated according to the manufacturer’s instructions for the Bac-to-Bac system, and transiently transfected into Sf9 insect cells in Sf900III medium (Gibco) with penicillin-streptomycin-amphotericin B (Fujifilm Wako) at 28 °C for 48 h. The cells were collected by centrifugation and disrupted by sonication in Tris-buffered saline (TBS, 20 mM Tri-HCl [pH 8.0] and 150 mM NaCl). Cell debris was removed by centrifugation. The His-tagged proteins were purified with TALON cobalt affinity resin (Clontech). The buffer was exchanged with TBS using a PD-10 desalting column (Cytiva). The tag was cleaved with TEV protease overnight at 4 °C and removed by passing through TALON cobalt affinity resin. ACE2 was purified by SEC using a Superdex200 Increase 10/300 column (Cytiva) in TBS. The peak fractions were collected and concentrated to 5 mg mL^−1^ using a Vivaspin Turbo 4 MWCO 50-kDa filter (Sartorius).

### Protein Expression and Purification of Tandem CeSPIACE for SPR Measurement.

The tandem CeSPIACE (two CeSPIACE sequences linked via GS linker) with an N-terminal 10xHis-enhanced green fluorescent protein tag linked through a thrombin recognition sequence and a C-terminal Avi tag linked through a 15-repeat GGS linker was subcloned into the pGEX4T plasmid. The proteins were expressed using *Escherichia coli* BL21 (DE3) (Novagene) in Luria Bertani medium with 25 µg mL^−1^ ampicillin. The cells were grown at 37 °C until absorbance at 600 nm reached 0.5, induced with 1 mM isopropyl 1-thio-β-D-galactopyranoside for 5 h, harvested by centrifugation, and disrupted by sonication in TBS containing 10 µg mL^−1^ DNase I, 1 µg mL^−1^ lysozyme and protease inhibitor cocktail (Roche). Cell debris was removed by centrifugation. The His-tagged proteins were purified with TALON cobalt affinity resin (Clontech). The tag was cleaved with thrombin (Fujifilm Wako) overnight at 4 °C. The tandem CeSPIACE was purified by SEC using a Superdex200 Increase 10/300 column (Cytiva) in TBS. The peak fractions were collected and concentrated to 2 mg mL^−1^ using an Amicon Ultra-4 MWCO 3-kDa filter (Merck Millipore).

### Crystallization and Data Collection.

The purified RBD/peptide complexes were crystallized by sitting drop vapor diffusion with a reservoir as follows: 0.1 M HEPES (pH 7.5) and 10% PEG 8000 for Ce9/WT RBD; 0.2 M magnesium formate dihydrate for Ce41/Alpha-type RBD; 0.08 M MES (pH 6.5), 0.16 M calcium acetate hydrate, 14.4% PEG 8000, and 20% glycerol for Ce59/Alpha-type RBD; 0.1 M BICINE (pH 9.0) and 20% PEG 6000 for Ce59/Delta-type RBD; 0.2 M sodium thiocyanate and 20% PEG 6000 for Ce149/BA.2-type RBD; 0.1 M BIS-Tris (pH 5.5), 0.1 M ammonium acetate, and 17% PEG 10000 for CeSPIACE/BA.2-type RBD; 0.1 M HEPES (pH 7.5), 10% PEG 6000, and 5% MPD for CeSPIACE/BA.5-type RBD; and 0.1 M CHES (pH 9.5) and 20% PEG 8000 for CeSPIACE/XBB.1.5-type RBD. Crystals appeared in drops at 20 °C and grew for 1 wk. The crystals were soaked in the same mother liquor with a gradually increasing concentration of glycerol from 0% to 18 % before being flash-frozen in liquid nitrogen. X-ray diffraction data were collected at the SPring-8 BL45XU beamline and the Swiss Light Source (SLS) X06DA and X06SA beamline. Data collection at SPring-8 was performed using the automatic data-collection system ZOO (43), and the data were automatically processed by KAMO (44). Data collection at SLS was performed via the remote access service, and the data were automatically processed by adp (45). Manual data processing using XDS (46) and truncation using the CCP4 program suite (47) were performed as necessary. The initial phase was determined by molecular replacement with Phaser (48) using the RBD structure (PDB code 7JZU) as the search template. The atomic model was rebuilt by manual model building in COOT (49) and refinement in PHENIX (50) and Refmac5 (51). The dihedral angles of the peptide bonds of all models were modified based on Ramachandran plots calculated with MolProbity (52). Structure data and refinement statistics are shown in *SI Appendix*, Table S1.

### Cryo-EM Sample Preparation and Data Collection.

The purified ectodomain/CeSPIACE complexes were diluted to 0.4 to 0.5 mg mL^−1^ with SEC running buffer, and 3.0-µL aliquots of the sample were applied to Quantifoil R1.2/1.3 300 mesh Au grids, blotted for 3 to 4 s at 4 °C, and plunge-frozen in liquid ethane using a Vitrobot Mark IV (Thermo Fisher Scientific). Data collection was performed on a JEM-Z320FHC (JEOL) electron microscope at 300 kV, cooled with liquid nitrogen, and equipped with an in-column energy filter using a zero-loss slit width of 20 eV. All images were recorded on a K2 Summit direct electron detector (Gatan) in the electron counting mode, using SerialEM (53). The calibrated pixel size was 0.96 Å on the specimen level, and 8-s exposures were dose-fractionated into 40 frames with an electron flux of 8 e − /pix/s.

For all datasets, image processing was performed with the RELION-4.0 (54, 55). Specimen movement was corrected using RELION-implemented motion correction; the contrast transfer function (CTF) parameters were estimated using CTFFIND4 (56), and images showing substantial ice contamination, abnormal backgrounds, or poor Thon rings were discarded.

For the initial dataset of the ectodomain with the BA.2-type RBD complex, particles were picked with Warp (57) using the pretrained BoxNet model, extracted with rescaling from 360 × 360 to 90 × 90 pixel images, and subjected to two-dimensional (2D) classification to select particles with fine structural details of the spike trimer. The selected particles were subjected to three-dimensional (3D) classification into four classes, three of which were selected and used to re-extract the corresponding particles into 360 × 360 pixel images. The re-extracted particles were refined with C1 symmetry to a resolution of 3.7 Å according to the Fourier shell correlation = 0.143 criterion (58). The refined particle coordinates were used to re-extract the corresponding particles into 600 × 600 pixel images with recentering on the Z = −80 pixel for a “downward” shift of the single spike trimer in the voxel, and rescaled into 160 × 160 pixel images. The re-extracted particles were subjected to 2D classification to select classes representing “docking dimer” structures of the spike trimers. The selected particles were subjected to 3D classification into four classes, one of which was selected as a training set to repick the docking dimer particles using the Topaz autopicking function (59) in RELION-4.0. Four datasets were collected for the ectodomain with the BA.2-type RBD complex, and the particles were autopicked using Topaz, extracted with rescaling from 600 × 600 to 160 × 160 pixel images, and subjected to 2D classification and 3D classification. The selected particles corresponding to the best class for each dataset were re-extracted into 600 × 600 pixel images without rescaling and subjected to 3D refinement, per-particle CTF refinement, and Bayesian polishing with rescaling from 510 × 510 to 340 × 340 pixels. The polished particles from all four datasets were combined and subjected to multiple rounds of 3D classification, 3D refinement, and per-particle CTF refinement. Final 3D refinement and postprocessing yielded a map at an overall resolution of 4.9 Å.

For the initial dataset of the ectodomain with the BA.5-type RBD complex, particles were picked with Gautomatch (https://www.mrc-lmb.cam.ac.uk/kzhang/Gautomatch/) with templates from the 2D class averages of the CeSPIACE-bound BA.2 spike trimers, extracted with rescaling from 360 × 360 to 120 × 120 pixel images, and subjected to 2D classification, and subsequently to 3D classification into five classes. Particles classified into three classes representing the “RBD 3 up” conformation were selected and re-extracted into 360 × 360 pixel images, then refined with C1 symmetry to a resolution of 4.8 Å. The refined particle coordinates were used to re-extract the corresponding particles into 600 × 600 pixel images with recentering on the Z = −80 pixel, rescaled into 160 × 160 pixel images, and subjected to 3D classification into four classes. Particle sets corresponding to the two best classes were separately selected and re-extracted with rescaling from 510 × 510 to 340 × 340 pixel images and refined with C1 symmetry to resolutions of 8.2 and 6.4 Å, respectively.

### SPR Measurements.

The Avi-tagged proteins were biotinylated by BirA biotin-protein ligase (Avidity). The buffer was exchanged with HBS-EP+ buffer (Cytiva) consisting of 0.01 M HEPES, 0.15 M NaCl, 0.003 M EDTA, and 0.05% (v/v) surfactant P20 using a PD-10 desalting column (Cytiva). The SPR measurements were performed with a Biacore S200 (Cytiva) using the Biotin CAPture Kit containing the Series S Sensor Chip CAP, the Biotin CAPture Reagent, and the Regeneration solution (Cytiva). Direct binding studies of WT and mutant RBDs to biotinylated peptides were conducted at 25 °C with HBS-EP+ buffer. The chip surface was first conditioned with three successive 60-s pulses of Regeneration solution (6 M guanidine HCl, 0.25 M NaOH) at a flow rate of 10 µL min^−1^, followed by three startup cycles using standard conditions for the Biotin CAPture Kit before the kinetic analysis. In each measurement cycle, Biotin CAPture Reagent was applied to the reference and active flow cells for 300 s at a flow rate of 2 µL min^−1^. Biotinylated peptides were injected onto the active flow cell at a flow rate of 10 µL min^−1^ to produce an immobilization level of 3.3 to 5.6 resonance units. Serial dilutions of WT and mutant RBDs were injected in HBS-EP+ at a flow rate of 30 µL min^−1^, with a contact time of 120 s and a dissociation time of 600 s. Three-fold serial dilutions of analyte proteins were analyzed in a multicycle kinetic program. After each analysis cycle, the sensor chip was regenerated with Regeneration solution for 120 s at a flow rate of 10 µL min^−1^. Three independent experiments were carried out. Data were collected using dual detection at 10 Hz and analyzed with the Biacore S200 Evaluation Software (version 1.1, Build 27; Cytiva). Double referencing was performed by first subtracting the analyte responses over the reference flow cell from the corresponding responses in the active flow cell, followed by subtraction of sensorgrams for 0-nM analyte RBDs from sensorgrams corresponding to all other concentrations of analyte RBDs. After double referencing, the data were fitted to a simple 1:1 Langmuir binding model to determine the association rate constant (k_on_), dissociation rate constant (k_off_), and Rmax (saturation signal where all functional ligand molecules bound to analyte molecules). K_D_ was calculated using the ratio of k_off_ to k_on_ (k_off_/k_on_).

Direct binding studies of CeSPIACE to biotinylated WT and mutant RBDs were conducted at 25 °C with HBS-EP+ buffer. The preparatory conditioning of the chip surface, the startup procedures, and the application of the Biotin CAPture Reagent were all performed according to the conditions described above. Biotinylated RBDs were injected onto the active flow cell at a flow rate of 10 µL min^−1^ to produce an immobilization level of approximately 170 resonance units. Serial dilutions of CeSPIACE were injected in HBS-EP+ at a flow rate of 30 µL min^−1^, with a contact time of 120 s and a dissociation time of 600 s. Two-fold serial dilutions of CeSPIACE were analyzed in a multicycle kinetic program. After each analysis cycle, the sensor chip was regenerated with Regeneration solution for 120 s at a flow rate of 10 µL min^−1^. All measurements were performed independently in triplicate.

Sensorgrams and 1:1 binding model curve fits were exported using the S200 Evaluation Software and replotted in GraphPad Prism (v9.1.0; GraphPad Software).

### Protein Peak-Shift Assay in FSEC.

The pFastBac1 plasmid described above with inserted mutant RBD (residues Arg319-Phe541) was transiently transfected into Expi293F cells using FuGENE 4 K (Promega). The cells were cultured in Expi293 expression medium (Gibco) with antibiotics and 5 mM sodium butyrate using a 12-well plate for 72 h at 37 °C. The RBD was harvested from the cell culture medium, purified with 0.2 mL of TALON resin and diluted in TBS to approximately 500 nM RBD, estimated by the Trp fluorescence (excitation/emission wavelength = 280/330 nm) of the RBD peak in FSEC. The mutant RBD was mixed with CeSPIACE or purified ACE2 at 1 μM or 500 nM, respectively. The mixture was analyzed using a Superdex200 Increase 5/150 column with detecting Trp fluorescence. The peak mobility by complex formation was calculated as follows.:

Peak mobility (%) = (RT_before_ - RT_after_) / (RT_before_ - RT_max_) × 100

RT_before_: Retention time before binding.

RT_after_: Retention time after binding, which reflects the equilibrium state between binding and dissociation according to the affinity.

RT_max_: Retention time when the complex is completely formed, using the Alpha-type RBD of the highest affinity.

### Cells and Viruses for Antiviral Assay.

All cell cultures were maintained at 37 °C in a 5% CO_2_ incubator. VeroE6/TMPRSS2 cells (34) were maintained in Dulbecco’s modified Eagle’s medium (DMEM, Nacalai Tesque) supplemented with 10% fetal bovine serum (FBS, Thermo Fisher Scientific), 100 units mL^−1^ penicillin, 100 μg mL^−1^ streptomycin (Nacalai Tesque), and 1 mg mL^−1^ G418 (Invivogen). Calu-3 (human lung adenocarcinoma, ATCC HTB-55) cells were cultured in Eagle’s minimum essential medium (Nacalai Tesque) supplemented with 10% FBS, penicillin, and streptomycin.

The SARS-CoV-2 variants Wuhan/D614G [OMC-510 (60)], Delta (OMPU-118a), Omicron BA.1 (OMPU-118a), Omicron BA.2 (OMPU-411), Omicron BA.5 (OMPU-715a), and Omicron XBB.1.5 (OMPU-620a) were isolated from patients who were diagnosed with COVID-19 at the Osaka Medical and Pharmaceutical University Hospital from May 2020 to June 2023. The nucleotide sequences of the spike gene of the clinical isolates were confirmed by sequencing analysis. SARS-CoV-2 Alpha (QK002) variants were kindly provided by the National Institute of Infectious Diseases, Japan. All isolates were propagated using Vero E6/TMPRSS2 cells.

### Plaque-Forming Assay.

Infectious titers of SARS-CoV-2 were determined by a plaque-forming assay. VeroE6/TMPRSS2 cells, seeded in 24-well plates at a density of 1.5 × 10^5^ cells/well 1 d before assay, were incubated with virus sample at 37 °C for 2 h, followed by cultivation with Eagle’s minimum essential medium containing 2% FBS and 1% methylcellulose (Fujifilm, Wako Pure Chemical Corporation). At 3 d after infection, cells were fixed with formaldehyde, and plaques were visualized by staining with 1% crystal violet. For titration of Omicron variants, formaldehyde-fixed VeroE6/TMPRSS2 cells were permeabilized with PBS containing 0.5% Triton X-100 and incubated with rabbit anti-SARS-CoV-2 nucleocapsid polyclonal antibody (GeneTex GTX135357, 1000-fold diluted in PBS) at 4 °C overnight. After three washes with PBS, cells were incubated with Alexa Fluor 488-conjugated anti-rabbit secondary antibody (Thermo Fisher Scientific, 1000-fold diluted in PBS) at 37 °C for 2 h. The plate was washed three times with PBS, and fluorescent plaques were detected using a Typhoon FLA 9000 scanner (GE Healthcare Life Sciences). Virus titers were calculated as PFU mL^−1^.

### Plaque Reduction Assay.

One hundred PFU of SARS-CoV-2 was mixed with serial dilutions of CeSPIACE in 200 μL reaction containing DMEM and subjected to a plaque-forming assay.

### ToA Assay.

Calu-3 cells in a 24-well plate (1 × 10^5^ cells/well for 1 d prior assay) were incubated with 200 μL DMEM mixture containing 10,000 PFU of SARS-CoV-2 and 100 nM CeSPIACE (preinfection treatment) or control buffer (postinfection, late-infection, and control treatments) in reaction containing DMEM at 37 °C. After 2 h incubation, cells were cultured with DMEM/10% FBS in the presence of 100 nM CeSPIACE (postinfection treatment) or control buffer (preinfection, late-infection, and control treatments). For the late-infection treatment condition, 100 nM CeSPIACE was added to the culture 18 h after infection. The culture supernatants were collected at 48 h after infection and subjected to the plaque-forming assay to measure virus titers.

### Antiviral Assay Using Hamsters.

Animal experiments were performed in an Animal Biosafety Level 3 (ABSL3) facility in accordance with the Guidelines for Animal Research of Osaka Medical and Pharmaceutical University, Faculty of Medicine. All procedures were approved by the Committee of Animal Use and Care of Osaka Medical Pharmaceutical University (Approval No. AM23-069). Female Syrian hamsters (purchased from Japan SLC) at 4 wk of age were intranasally infected with 5000 PFU of the SARS-CoV-2 Delta variant (in 20 μL DMEM) under 2% isoflurane inhalation anesthesia. At 2 h after inoculation, hamsters were intratracheally administered 100 μL of 100 μM CeSPIACE or basal buffer (50 mM HEPES, pH 8.0) using an aerosol sprayer (Natsume Seisakusho Co. Ltd.) under 2% isoflurane anesthesia. Inhaled administration of CeSPIACE or control buffer was repeated daily until 3 d after infection, and the body weights of the hamsters were monitored daily. At 4 d after infection, hamsters were humanely killed by anesthesia with 4% isoflurane and intramyocardial injection of potassium chloride (> 1 mmol kg^−1^), and lung tissues were collected. The right lung was fixed in 10% formaldehyde and subjected to histopathologic analysis. The left lung was homogenized in 500 μL DMEM using a Precellys Lysing Kit (Bertin Technologies) and a TissueLyser II (Qiagen). Total RNA was isolated from 100 μL of the left lung homogenate using a MagMAX *mir*Vana Total RNA Isolation Kit (Thermo Fisher Scientific), and quantitative reverse transcription PCR analysis was performed using an iTaq Universal Probes One-Step Kit (Bio-Rad) and a QuantStudio 3 Real-Time PCR System (Thermo Fisher Scientific). In another experiment, the supernatant of the homogenate was serially diluted 10-fold (10^−1^ to 10^−8^ dilutions) with DMEM, and 200 μL of the dilutions were subjected to plaque forming assay. Viral RNA was quantified using a standard curve of serial dilutions of SARS-CoV-2 N gene control RNA (Nippon Gene Co., LTD.) and the CDC 2019-nCoV_N1 primer and probe set (61). Relative quantification of cytokine genes (Ifng, Il6, Il10, and Cxcl10) was performed using a serial dilution of hamster total RNA and primers and probe sets as described previously (62).

### Histopathologic Analysis.

The right lung specimens were fixed in 10% neutral-buffered formalin overnight at room temperature and embedded in paraffin wax. The formaldehyde-fixed and paraffin-embedded lung sections on glass slides were deparaffinized and hydrated for hematoxylin and eosin staining. For immunohistochemical analysis, the formaldehyde-fixed and paraffin-embedded lung sections on glass slides were deparaffinized and hydrated, then stained with a primary antibody against SARS-CoV/SARS-CoV-2 Nucleocapsid (cat. no. 40143-R001, Sino Biological Inc., Wayne, PA) at a 1:20,000 dilution with BOND Polymer Refine Detection (cat. no. DS9800; Leica Microsystems, Tokyo, Japan) and BOND-MAX (Leica Microsystems) according to the manufacturer’s instructions, in which staining was performed using 3,3’-diaminobenzidine tetrahydrochloride hydrate to visualize the SARS-CoV/SARS-CoV-2 Nucleocapsid via a brown precipitate. Hematoxylin and eosin and immunohistochemical-stained sections were evaluated using the VS120 Virtual Slide Microscope (Evident Corporation/Olympus, Tokyo, Japan) with OlyVIA software (Ver.2.9.1, Evident Corporation/Olympus) and a light microscope (Olympus BX43, Evident Corporation/Olympus) at 20× to 400× magnification.

### Statistical Analysis.

The mean values were obtained using a representative set of at least three independent experiments and are shown with error bars indicating the SD in the figures. Where indicated in the figures, statistical significance was determined using GraphPad Prism Software (GraphPad Inc.), and *P* values below 0.05 were considered significant [*, *P* < 0.05; **, *P* < 0.01; ***, *P* < 0.001; ns (not significance), *P* > 0.05].

## Supplementary Material

Appendix 01 (PDF)

## Data Availability

Atomiccoordinates have been deposited in the Protein Data Bank with Accession codes 8YDP (63), 8YDQ (64), 8YDR (65), 8YDS (66), 8YDT (67), 8YDU (68), 8YDV (69), 8YDW (70), 8YDX (71), 8YDY (72), and 8YDZ (73). Cryo-EMmaps have been deposited in the Electron Microscopy Data Bank with Accessioncodes EMD-39184 (74), EMD-39185 (75), and EMD-39186 (76).
