# Supplementary material for: Structure-guided engineering of a mutation-tolerant inhibitor peptide against variable SARS-CoV-2 spikes
Source: Proc Natl Acad Sci U S A. 2025 Jan 24;122(4):e2413465122. doi: 10.1073/pnas.2413465122 (PMC11789008; doi:10.1073/pnas.2413465122)
Supplement: Supplementary file 1 — Appendix 01 (PDF) [file pnas.2413465122.sapp.pdf]

Supporting Information for

Structure-guided engineering of a mutation-tolerant inhibitor peptide against variable SARS-CoV-2 spikes

Shun Nakamura, Yukihiro Tanimura, Risa Nomura, Hiroshi Suzuki, Kouki Nishikawa, Akiko Kamegawa, Nobutaka Numoto, Atsushi Tanaka, Shigeru Kawabata, Shoichi Sakaguchi, Akino Emi, Youichi Suzuki, Yoshinori Fujiyoshi*

* corresponding author: Yoshinori Fujiyoshi
Email: yoshi.cesp@tmd.ac.jp

This PDF file includes:

Supporting text
Figures S1 to S11
Tables S1 to S3

A

Peptide	Amino acid sequence
	1 10 20 30
Ca1	DKEWILQKIYEIMRLLDELGHAEASMRVSDLIYEFMKKG
Ce9	DKEWILQKIYEIMRRLDEEGHAEASMRVSDLIYEFMKKD
Ce41	DKEWILQKIYEIMRRLDEEGHGEASLRVSDLIYEFMKKD
Ce59	DKEWILQKIYEIMRRLDEEGHGEASLRVSDLIYEFMKRD
Ce149	DKEWILYKIYEIMVRLDEEGHGEASLMVSDLIYEFMKRD
CeSPIACE	DKLWILQKIYEIMVRLDEEGHGEASLMVSDLIYEFMKRD

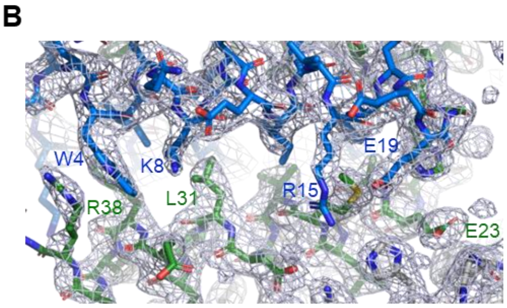

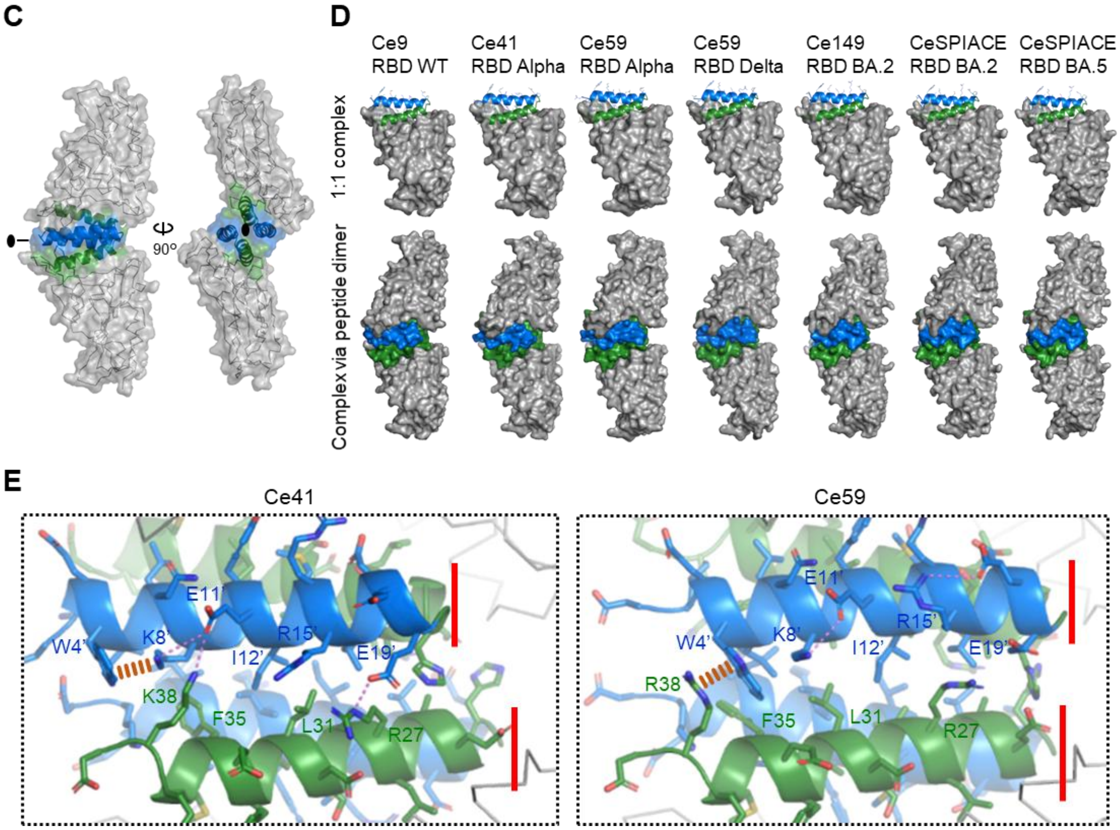

Fig. S1. Crystal structures of CeSPIACE or intermediate-stage peptides complexed with RBDs. (A) Amino acid sequences of CeSPIACE and intermediate-stage peptides. Differences among the sequences are shown in red. (B) Representative electron density map of the CeSPIACE-RBD XBB.1.5 crystal structure. (C) Overall structure of the CeSPIACE-RBD XBB.1.5 complex. Black ovals indicate the 2-fold axis. Colors correspond to those used in Fig.1. (D) Overall structures of CeSPIACE or intermediate-stage peptides complexed with RBDs. (E) Comparison of dimer configuration between Ce41 and Ce59 complexed with the Alpha-type RBD. The only difference between Ce41 and Ce59 is the 38th residue (K or R). Red bar indicates the position of the turn. Salt bridges and cation-pi interactions are indicated by magenta dots and brown oval dots, respectively.

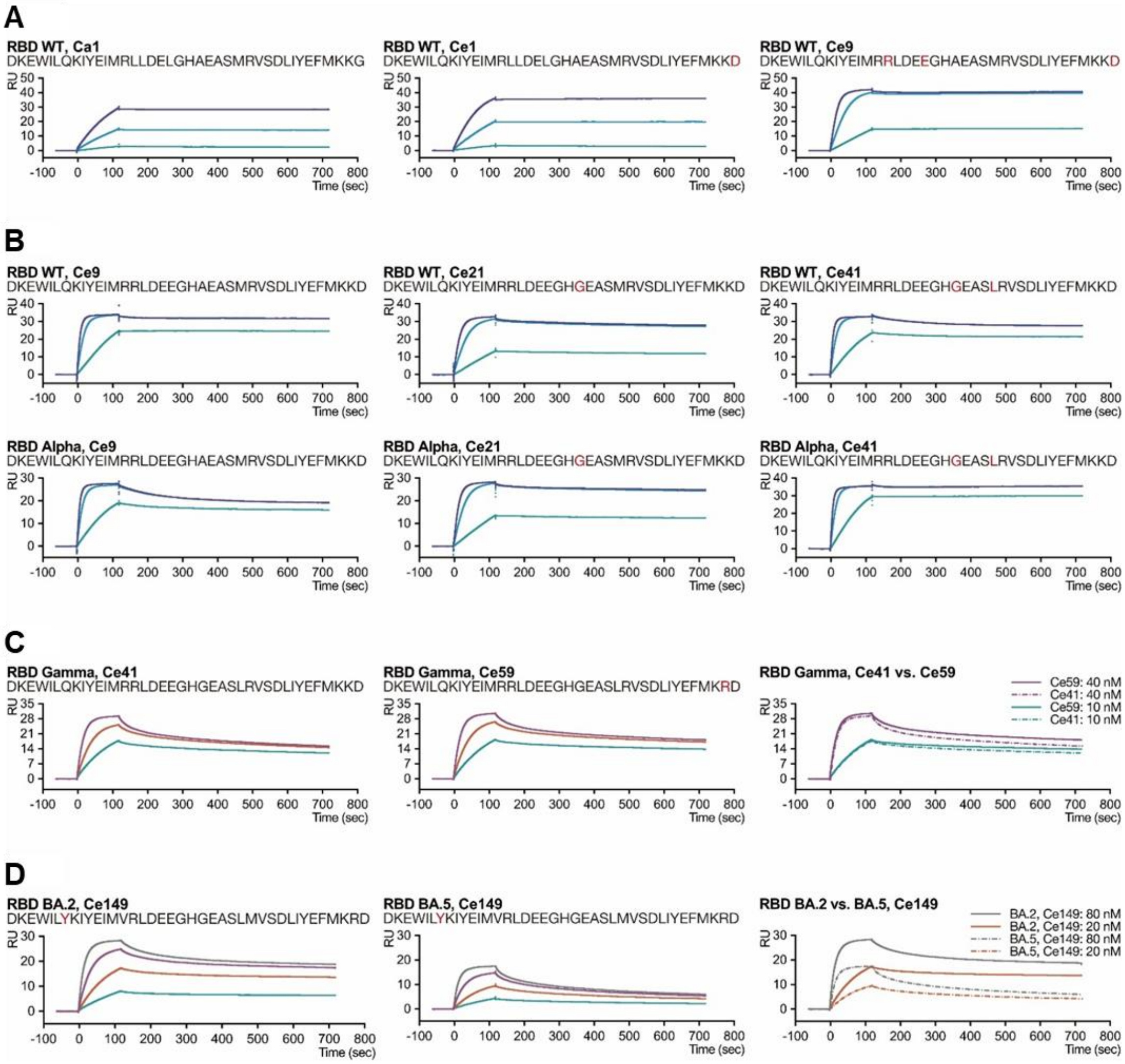

Fig. S2. SPR analyses for optimizing the peptide sequence. A binding assay using SPR was performed to optimize a peptide sequence with strong binding activity to mutant RBDs. The peptide sequences are shown above the sensorgrams, and the differences in the sequences are indicated in red. SPR measurements were performed using HBS-EP+ as the running buffer in (A), (C), and (D), and HBS-EP+ containing 0.1% DMSO in (B). (A) Comparisons among Ca1, Ce1, and Ce9 using a sensor chip immobilized wild-type RBD. Peptide concentrations were prepared stepwise, 10, 50, and 100 nM. R15, E19, and D39 improved the binding rate. (B) Comparisons among Ce9, Ce21, and Ce41 using a sensor chip immobilized wild-type RBD or Alpha-type RBD. Peptide concentrations were prepared stepwise, 10, 50, and 100 nM. G22 and L26 improved the dissociation rate against N501Y mutation. (C) Comparison between Ce41 and Ce59 using a sensor chip immobilized Gamma-type RBD. Peptide concentrations were prepared stepwise, 10, 20, and 40 nM. R38 improved the dissociation rate. (D) Sensorgrams of Ce149 using a sensor chip immobilized BA.2-type RBD or BA.5-type RBD. Peptide concentrations were prepared stepwise, 10, 20, 40, and 80 nM. Y7 is the only difference in the CeSPIACE sequence and affects the dissociation from the BA.5-type RBD with the F486V mutation.

A

	339	346	371	373	375	376	405	408	417	440	446	452	477	478	484	490	493	496	498	501	505
Wild type	G	R	S	S	S	T	D	R	K	N	G	L	S	T	E	F	Q	G	Q	N	Y
Alpha																				Y	
Beta									N						K					Y	
Gamma									T						K					Y	
Delta												R		K							
Epsilon												R									
Zeta															K						
Theta															K					Y	
Eta															K						
Iota													N		K						
Kappa												R			Q						
Labmda												Q			K	S				Y	
Mu		K																			
Omicron BA.1	D		L	P	F				N	K	S		N	K	A		R	S	R	Y	H
Omicron BA.2	D		F	P	F	A	N	S	N	K			N	K	A		R		R	Y	H

B

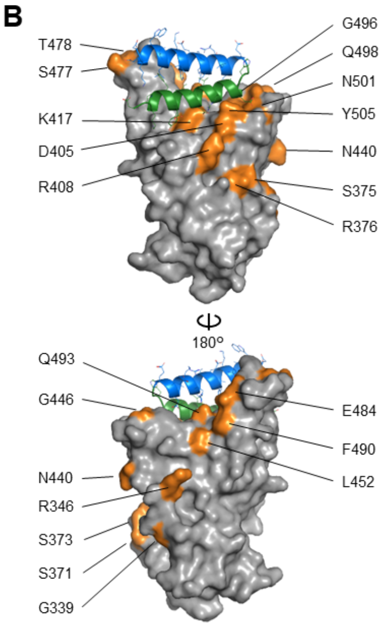

C

	339	346	356	368	403	444	445	446	450	452	456	460	478	481	483	484	486	490	493	521
BA.2	D	R	K	L	R	K	V	G	N	L	F	N	K	N	V	A	F	F	R	P
BA.2.3.20						R			D	M		K				R				
BA.2.10.4								S									P		Q	
BJ.1	H	T		I			P	S							A			V		
BA.2.12.1										Q										
BA.2.75	H							S				K							Q	
BA.2.75.2	H	T						S				K					S		Q	
CH.1.1	H	T				T		S		R		K					S		Q	
BM.1.1	H	T						S				K					S		Q	
BM.1.1.1	H	T						S				K					S	S	Q	
BN.1	H	T	T					S				K						S	Q	
BN.2.1	H		T					S				K						S	Q	
BR.1	H					M		S		R		K							Q	
BR.2	H	T						S		R		K					I		Q	
BL.1	H	T						S				K							Q	
CA.1	H	T						S		R		K					S		Q	
XBB.1	H	T		I			P	S				K					S	S		
XBB.1.5	H	T		I			P	S				K					P	S		
XBB.1.9.1	H	T		I			P	S				K					P	S		
XBB.1.9.2	H	T		I			P	S				K					P	S		
EG.5	H	T		I			P	S			L	K					P	S		
XBB.1.16	H	T		I			P	S				K	R				P	S		
XBB.2.3	H	T		I			P	S				K					S	S		S
XBF	H	T						S		R		K					P	S	Q	
BA.2.86	H		T		K		H	S	D	W		K		K	-	K	P		Q	
BA.3								S											Q	
BA.4										R							V		Q	
BA.4.6		T								R							V		Q	
BA.4.6.1		T								R							V		Q	
BA.5										R							V		Q	
BA.5.2		T				T				R							V		Q	
BE.1.1		I								R							V		Q	
BQ.1						T				R		K					V		Q	
BQ.1.1		T				T				R		K					V		Q	
BU.1						M				R		K					V		Q	
BA.5.6.2						T				R							V		Q	
BF.7		T								R							V		Q	

D

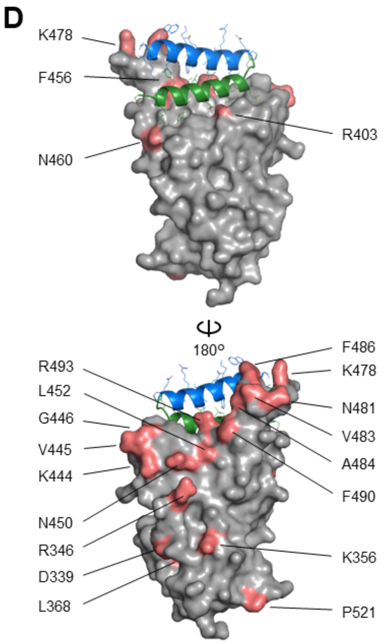

Fig. S3. RBD mutations of VOCs, VOIs, and VUMs. The lists are based on information released by the WHO. (A) RBD mutation sites of WHO-labeled variants from Alpha to Omicron. Mutations against wild-type are shown in orange. Bars above numbers indicate CeSPIACE-binding sites. (B) Mutation mapping on wild-type RBD structure in complex with the peptide. RBD mutations of WHO-labeled variants are shown in orange. (C) RBD mutation sites of Omicron subvariants that have ever been under WHO surveillance, including VOCs, VOIs, and VUMs. Mutations against BA.2 are shown in pink. Bars above numbers indicate CeSPIACE binding sites. (D) Mutation mapping on BA.2-type RBD structure in complex with the peptide. RBD mutations of Omicron subvariants are shown in pink.

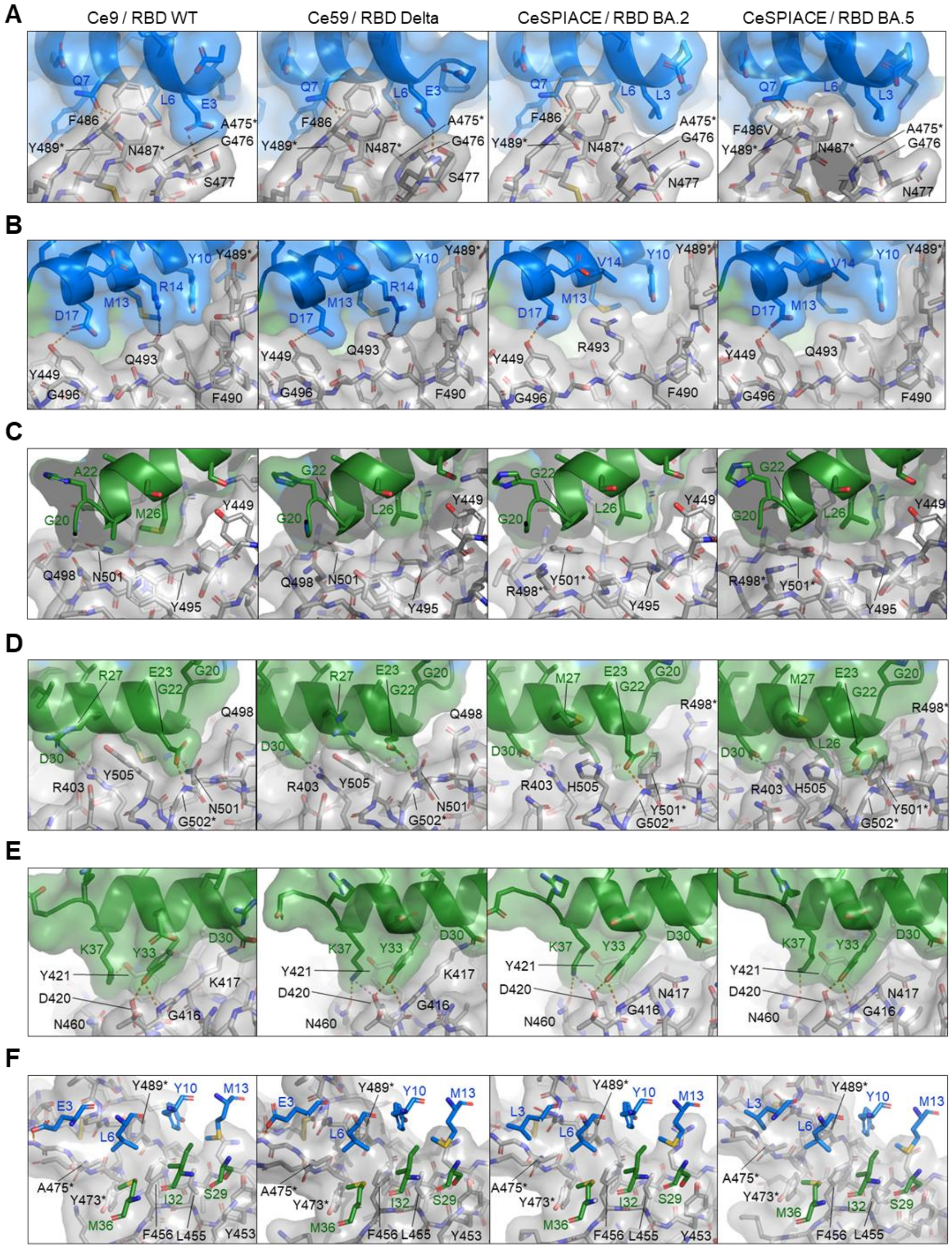

Fig. S4. Peptide-RBD interactions in the crystal structures. (A-F) Views in (A), (B), (C), (D), (E), and (F) are from the same angle as I, II, III, IV, V, and VI of Fig.1D, respectively.

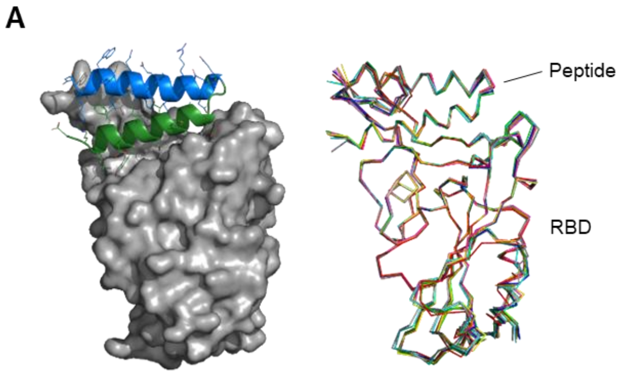

	RMSD (vs CeSPIACE / RBD XBB.1.5) RBD alone	Peptide / RBD
Ce9 / RBD WT	0.54	0.55
Ce41 / RBD Alpha	0.50	0.54
Ce59 / RBD Alpha	0.33	0.34
Ce59 / RBD Delta	0.37	0.38
Ce149 / RBD BA.2	0.24	0.23
CeSPIACE / RBD BA.2	0.26	0.25
CeSPIACE / RBD BA.5	0.45	0.40

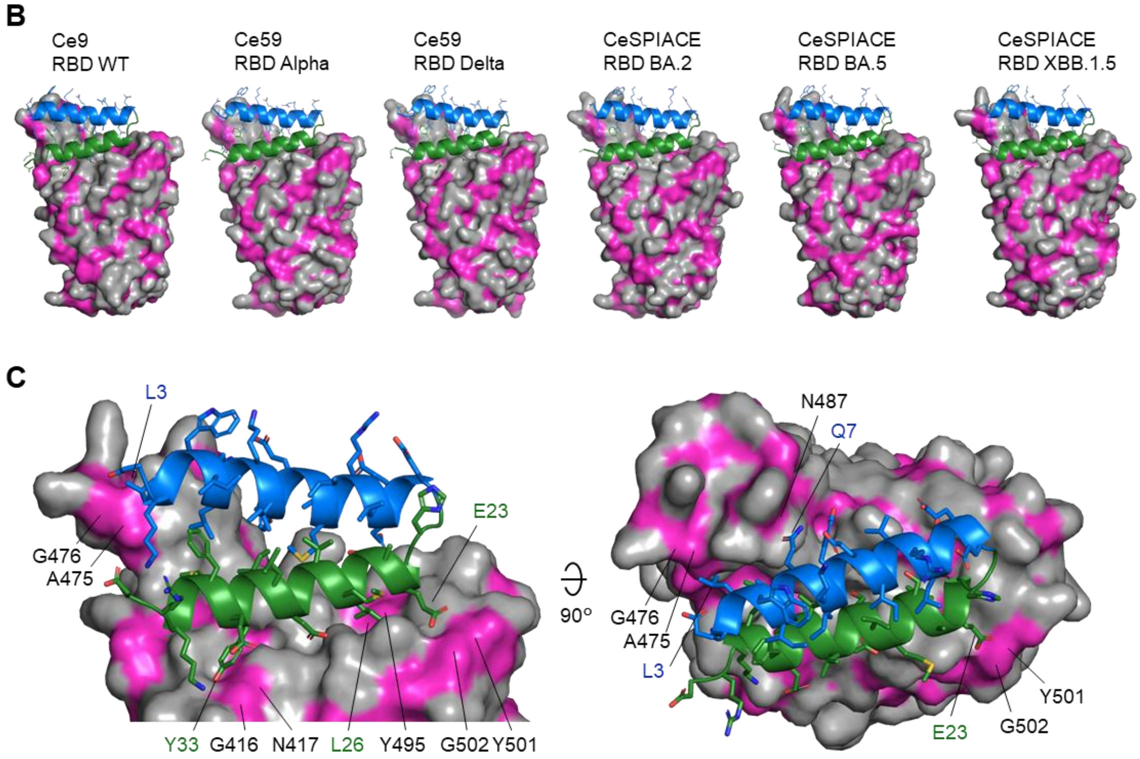

Fig. S5. Main-chain structure of RBD. (A) Superposition of the peptide-RBD complex structures. The left figure is the CeSPIACE-RBD XBB.1.5 structure, shown as a guide. Ribbon models of Ce9/RBD WT, Ce41/RBD Alpha, Ce59/RBD Alpha, Ce59/RBD Delta, Ce149/RBD BA.2, CeSPIACE/RBD BA.2, CeSPIACE/RBD BA.5 and CeSPIACE/RBD XBB.1.5 are shown in gray, cyan, blue, green, yellow, orange, red and magenta, respectively. RMSDs of the complex structures are shown on the right. (B) Main-chain structures on RBD surfaces. Main-chain atoms are shown in magenta. (C) Interactions of CeSPIACE with RBD main chain. The XBB.1.5-type RBD is shown as a representative.

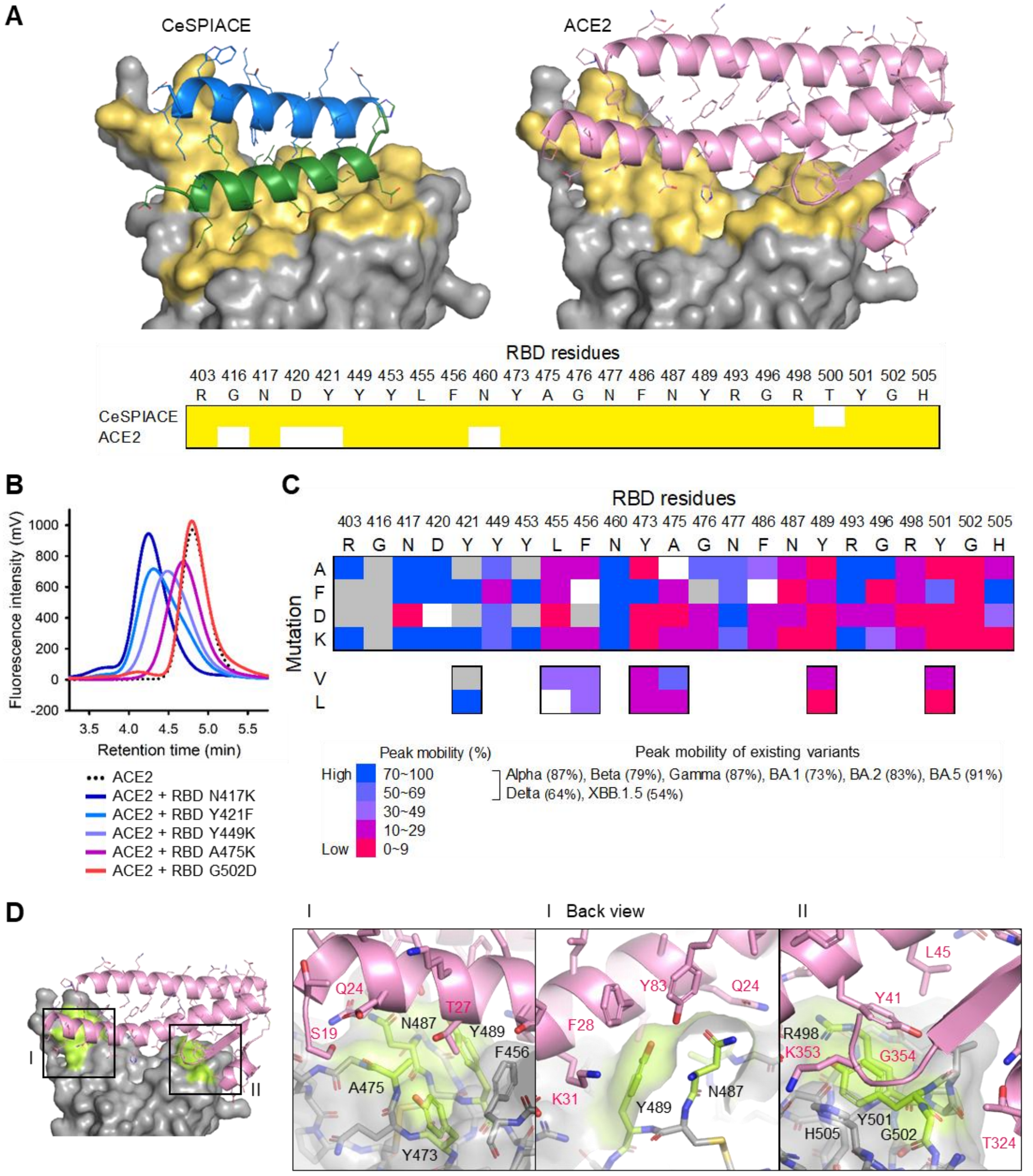

Fig. S6. RBD-ACE2 binding mechanism. (A) Binding region of RBD to CeSPIACE (left) and ACE2 (right). BA.2-type RBD is shown in gray. Binding regions on RBD are shown in yellow. CeSPIACE is shown as the same as Fig.1. ACE2 (PDB code 7XB0) is shown in pink. (B) Representative chromatogram of the peak-shift assay. The peak mobility was calculated using the retention time of ACE2 before and after RBD binding. (C) ACE2-affinity heatmap of RBD mutations on CeSPIACE binding site. The affinities are colored according to the peak mobility as shown in the legend. Unexpressed mutants are in gray, indicating that the folding was affected. The mobility of existing variants was >50%, so we estimated that mutations with mobility <30% rarely occur. (D) ACE2-RBD binding in the crystal structure (PDB code 7XB0). Critical RBD residues for ACE2 binding are highlighted in greenish yellow. Boxed areas with Roman numerals are enlarged on the right.

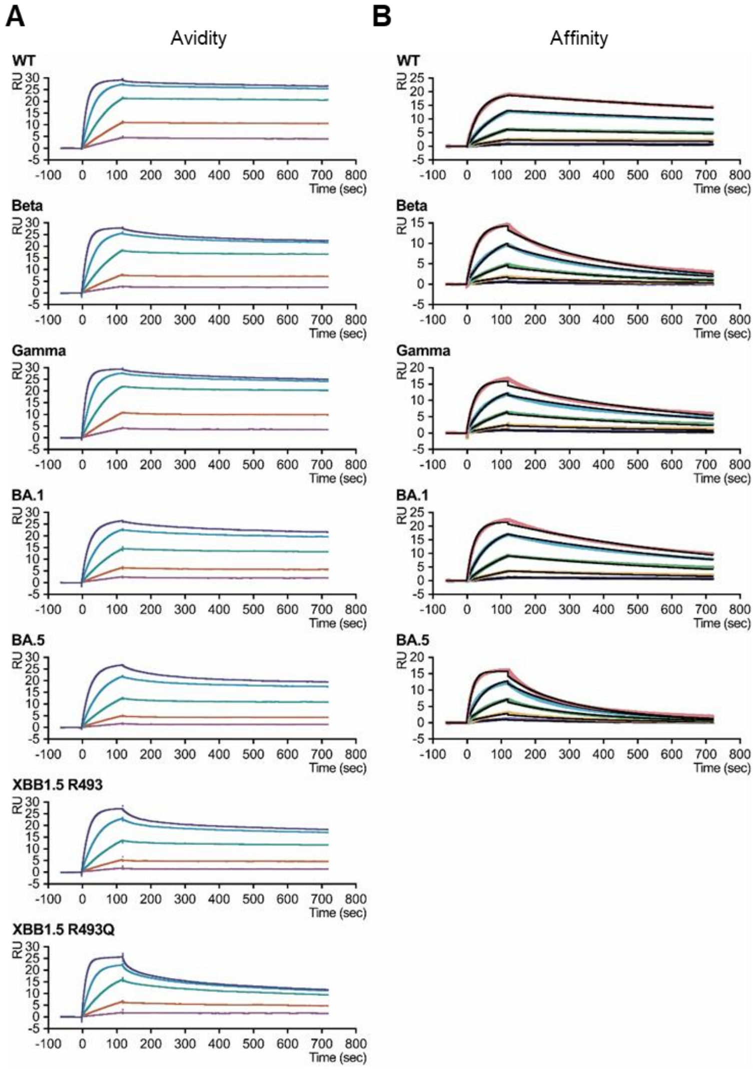

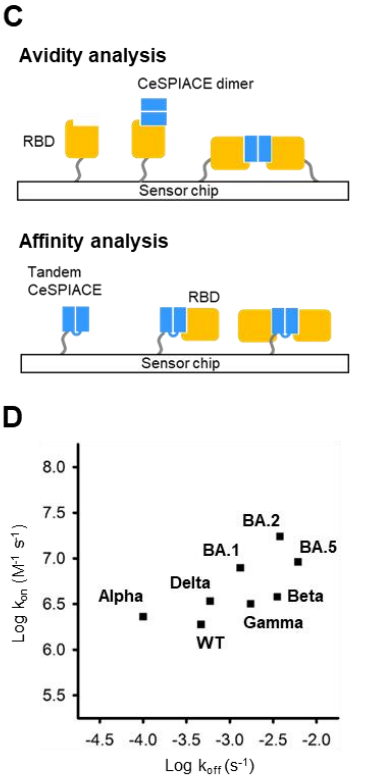

RBD type	k_{on} ($M^{-1}s^{-1}$)	k_{off} (s^{-1})
WT	$1.89 \pm 0.01 \times 10^6$	$4.66 \pm 0.01 \times 10^{-4}$
Alpha	$2.30 \pm 0.20 \times 10^6$	$9.99 \pm 0.60 \times 10^{-5}$
Beta	$3.81 \pm 0.24 \times 10^6$	$3.53 \pm 0.09 \times 10^{-3}$
Gamma	$3.20 \pm 0.10 \times 10^6$	$1.74 \pm 0.06 \times 10^{-3}$
Delta	$3.42 \pm 0.15 \times 10^6$	$5.92 \pm 0.03 \times 10^{-4}$
Omicron		
BA.1	$7.90 \pm 0.17 \times 10^6$	$1.32 \pm 0.01 \times 10^{-3}$
BA.2	$1.74 \pm 0.04 \times 10^7$	$3.80 \pm 0.05 \times 10^{-3}$
BA.5	$9.17 \pm 0.35 \times 10^6$	$6.11 \pm 0.12 \times 10^{-3}$

Fig. S7. SPR analysis of CeSPIACE-RBD binding. (A) SPR measurements of the avidity of CeSPIACE to mutant RBDs. CeSPIACE concentrations were prepared stepwise, 5, 10, 20, 40, and 80 nM. Regarding XBB.1.5, two RBD mutants with R493 or Q493 were evaluated. (B) SPR measurements of the affinity of CeSPIACE to mutant RBDs. The best fit of the data to the 1:1 binding model is shown by the black line. (C) Schematic diagram of a sensor chip. A sensor chip containing immobilized RBD for the avidity measurement is shown at the top. The CeSPIACE dimer may bridge two RBDs on a chip. A sensor chip for the affinity measurement is shown at the bottom, immobilizing tandem CeSPIACE, which allows a 1:1 binding model. (D) k_{on}- k_{off} rate map. Kinetic parameters were calculated from the fitting curves of three independent experiments.

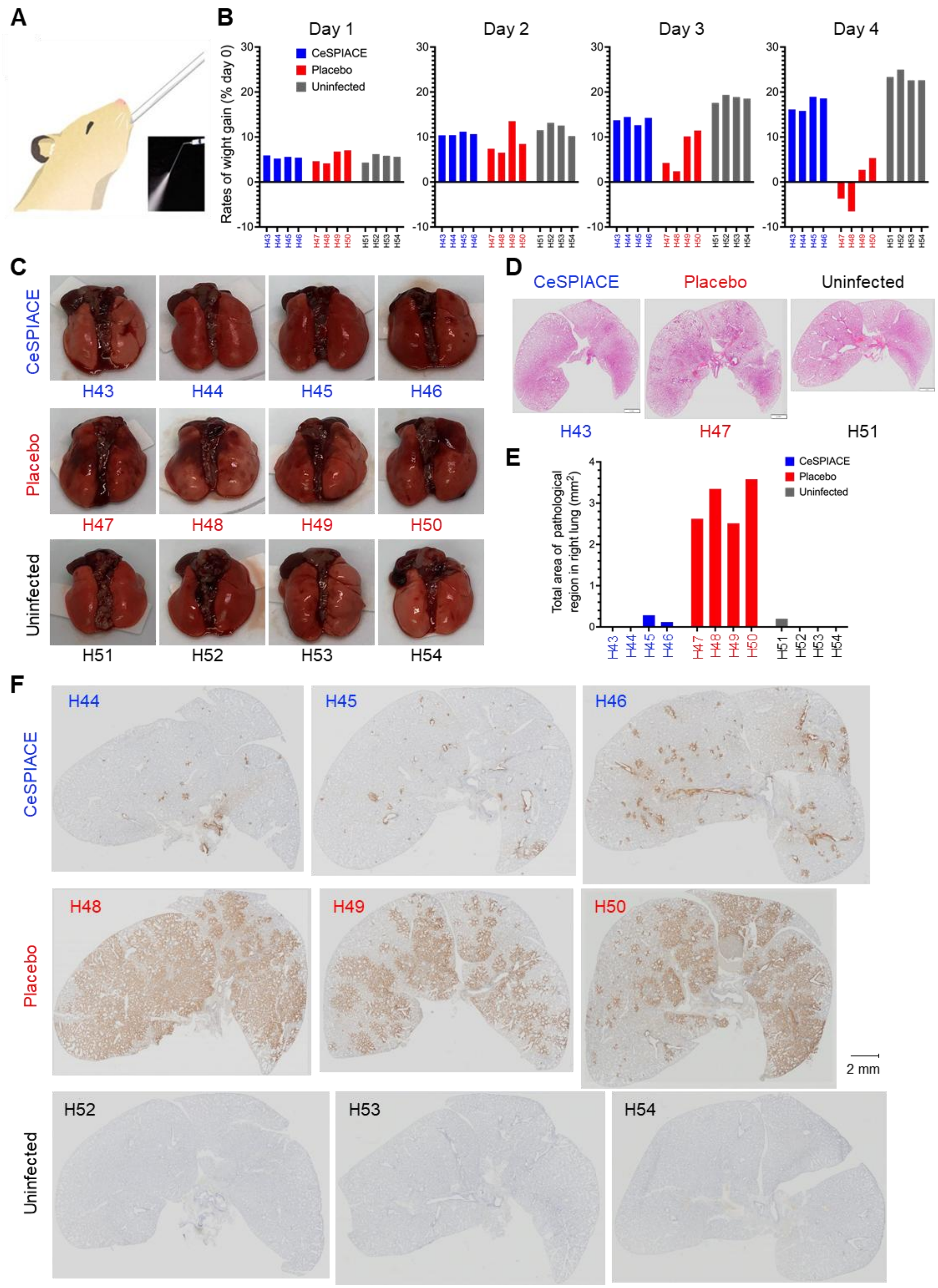

Fig. S8. Administration of CeSPIACE to a Syrian hamster model. (A) Schematic experimental details showing delivery of the medication to Syrian hamsters by bronchial spray (inserted figure: Nazume Co. Ltd.). Although the needle can be fully inserted into the bronchus, we only sprayed

the medication into the oral pharynx rather than placing the needle into the hamster's bronchus. (B) Rates of body weight gain (%) of hamster numbers H43-H54 from day 1 to day 4 compared with those at day 0. (C) Lung images of the hamsters at 4 days post-infection. (D) Pathologic anatomy of lung sections. (E) Summed total areas of pathologic lesions of the lungs administered CeSPIACE were very small, similar to those of lungs with no inoculation, whereas those of placebo-treated lungs were significantly larger. (F) Immunostaining images of lung sections of hamsters at 4 days post-infection. Pathologic anatomic sections were stained by anti-SARS-CoV-2 nucleocapsid antibody.

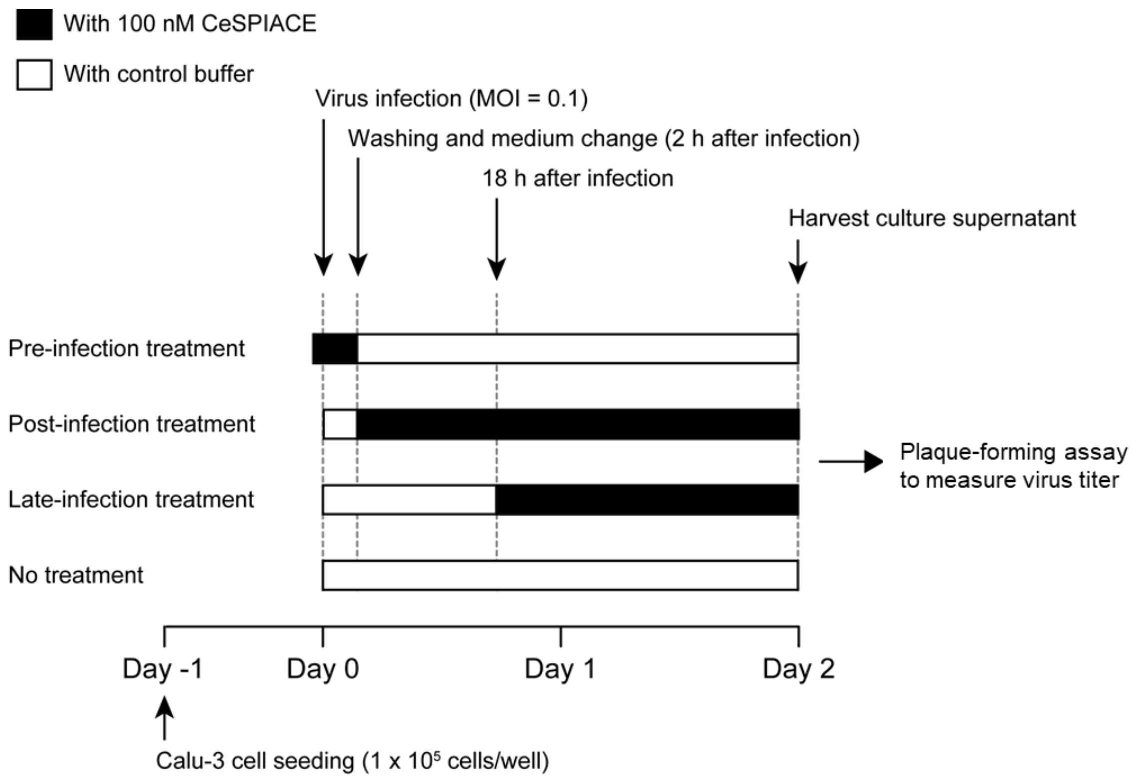

Fig. S9. ToA assay using Calu-3 cells. Experimental design of the ToA assay. At day -1, Calu-3 cells were seeded. Each virus type (wild-type, Alpha, Delta, or Omicron BA.5) was applied to the Calu-3 cells, and cell mediums were exchanged after 2 h. 100 nM CeSPIACE solutions were applied at the same time as virus infection (pre-infection), 2 h after infection (post-infection) or 18 h after infection (late-infection). After day 2, plaque-forming assays were performed using culture supernatants.

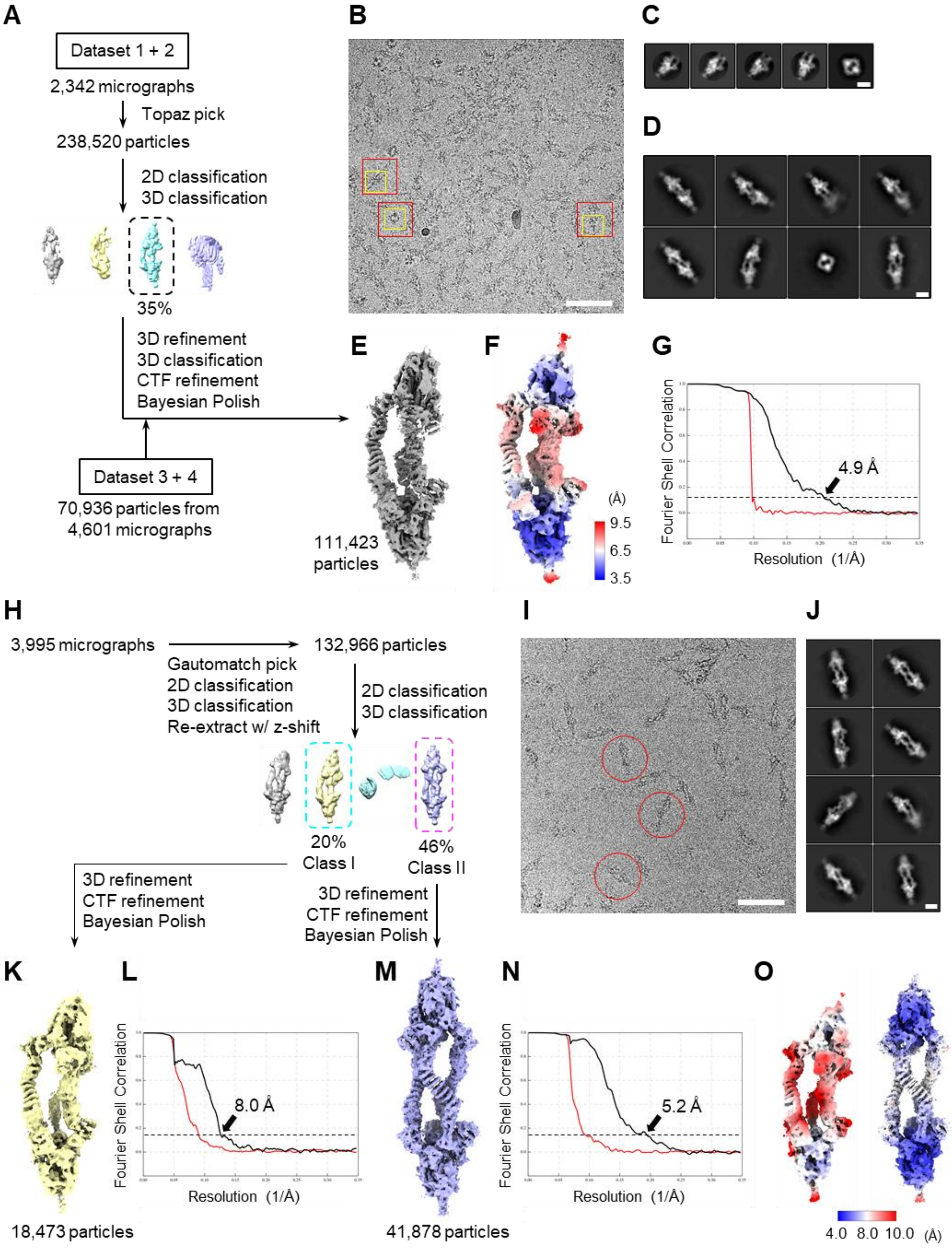

Fig. S10. Cryo-electron microscopy (EM) and image processing. (A) Cryo-EM data-processing workflow of the spike ectodomain (BA.2)-CeSPIACE complex. (B) Representative cryo-EM micrograph of the vitrified ectodomain (BA.2)-CeSPIACE complex. Yellow and red boxes indicate examples of the spike trimer and the 'docked dimer' of the trimers, respectively. Scale bar is 50 nm. (C) Selected two-dimensional (2D)-class averages of the spike-CeSPIACE complex trimer. (D) Selected 2D class averages of the 'docked dimer' of the spike-CeSPIACE complex. Scale bars are 10 nm. (E, F) The refined cryo-EM map (E) and local resolution map (F) of the

‘docked dimer’ of the spike-CeSPIACE complex. (G) Gold-standard Fourier shell correlation (FSC) of the refined map shown in (E) after correction for masking effects (black line). The phase-randomized FSC curve is colored red. The map resolution was estimated based on the FSC = 0.143 criterion. (H) Cryo-EM data-processing workflow of the spike ectodomain (BA.5)-CeSPIACE complex. (I) Representative cryo-EM micrograph of the vitrified ectodomain (BA.5)-CeSPIACE complex. Red circles indicate examples of the ‘docked dimer’ of the trimers. Scale bar is 50 nm. (J) Selected 2D class averages of the ‘docked dimer’ of the spike-CeSPIACE complex. Scale bars are 10 nm. (K. L) The cryo-EM map (K) of the spike-CeSPIACE complex refined from the class I, and gold-standard FSC curves (L) of the refined map after correction for masking effects (black line). (M, N) The cryo-EM map (M) of the spike-CeSPIACE complex refined from the class II, and gold-standard FSC curves (N) of the refined map after correction for masking effects (black line). The phase-randomized FSC curves are colored red. The map resolutions were estimated based on the FSC = 0.143 criterion. (O) Local resolution for the cryo-EM maps refined from the class I (left) and class II (right).

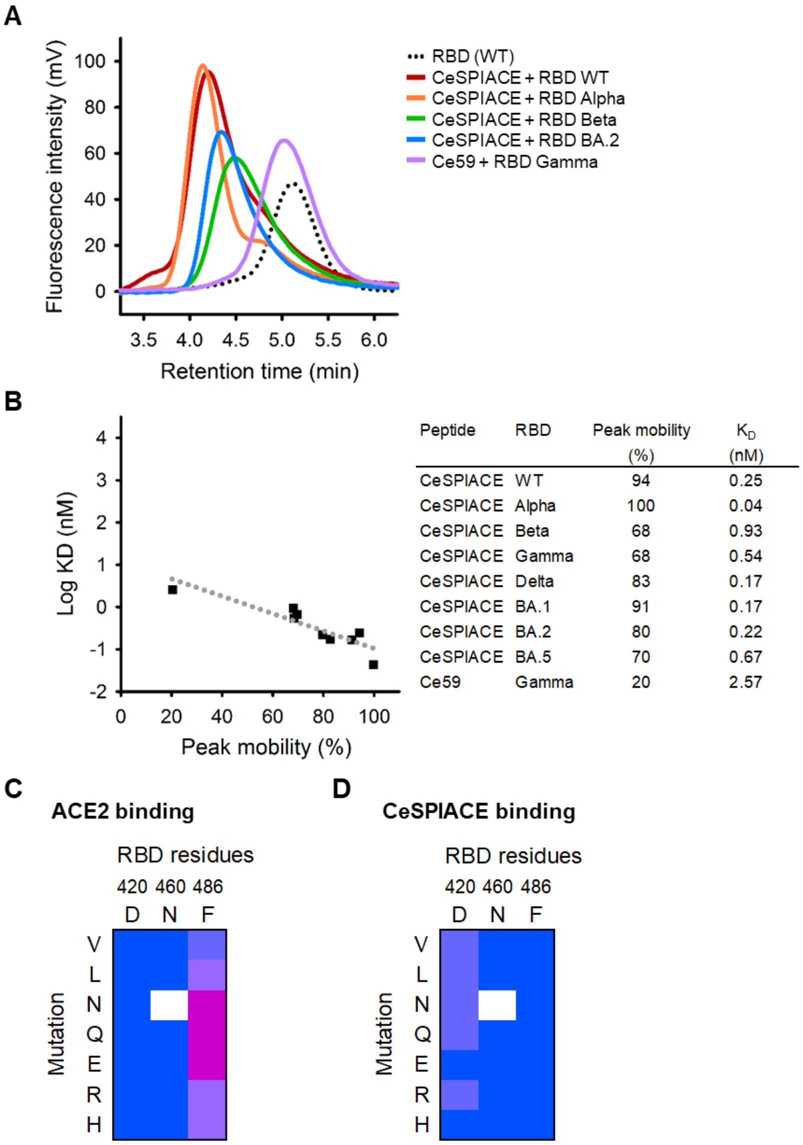

Peptide	RBD	Peak mobility (%)	K_D (nM)
CeSPIACE	WT	94	0.25
CeSPIACE	Alpha	100	0.04
CeSPIACE	Beta	68	0.93
CeSPIACE	Gamma	68	0.54
CeSPIACE	Delta	83	0.17
CeSPIACE	BA.1	91	0.17
CeSPIACE	BA.2	80	0.22
CeSPIACE	BA.5	70	0.67
Ce59	Gamma	20	2.57

Fig. S11. CeSPIACE affinity of RBD mutants. (A) Chromatograms of the peak-shift assay in binding of the peptide to representative mutant RBDs. Peak mobility is calculated using the retention time of RBD before and after the peptide binding. (B) Correlation between the peak mobility of FSEC and K_D of SPR. The peak mobility and K_D were each calculated from three independent experiments. (C) ACE2 affinity heatmap of mutant RBDs. The affinity is represented by the same color used in Fig. S5. (D) CeSPIACE affinity heatmap of mutant RBDs. The affinity is represented by the same color used in Fig. 5.

Table S1. Crystallographic data collection and refinement statistics.

	Ce9/ wild-type RBD (PDB 8YDP)	Ce41/ Alpha-type RBD (PDB 8YDT)	Ce59/ Alpha-type RBD (PDB 8YDR)	Ce59/ Delta-type RBD (PDB 8YDS)
**Data collection**				
Space group	$P3_1$	$P3_1$	$P3_121$	$P3_121$
Cell dimensions				
a, b, c (Å)	72.3, 72.3, 100.2	73.3, 73.3, 99.8	73.7, 73.7, 99.8	74.1, 74.1, 101.0
α, β, γ (˚)	90, 90, 120	90, 90, 120	90, 90, 120	90, 90, 120
Resolution (Å)	2.30 (2.44-2.30)	2.46 (2.61-2.46)	1.92 (2.04-1.92)	1.82 (1.93-1.82)
No. of reflections	26011 (4174)	21629 (3501)	23874 (3814)	29186 (4499)
R_{sym}	0.151 (2.108)	0.082 (1.477)	0.055 (1.274)	0.060 (0.450)
CC1/2	99.9 (55.6)	99.8 (76.4)	99.9 (79.6)	99.9 (90.0)
$I/\sigma I$	13.81 (1.26)	13.65 (1.14)	14.49 (1.66)	34.19 (6.98)
Completeness (%)	99.9 (99.5)	99.9 (99.7)	97.3 (95.1)	99.4 (96.6)
Redundancy	10.6 (10.7)	10.4 (10.0)	4.7 (4.7)	12.5 (8.9)
**Refinement**				
Resolution (Å)	39.1-2.3	39.2-2.5	39.3-2.3	39.7-1.9
No. of reflections	25961	20669	14157	25772
R_{work}/ R_{free} (%)	21.2/26.3	22.9/26.8	22.2/26.6	18.6/22.6
No. of atoms	4018	3806	1940	2229
Average B-factor (Å^2)	52.0	87.0	51.0	29.0
RMSD				
Bond lengths (Å)	0.00	0.01	0.00	0.01
Bond angles (˚)	0.42	1.01	0.52	0.74
Ramachandran				
Favored (%)	94.21	90.04	96.10	97.42
Allowed (%)	5.36	9.96	3.90	2.58
Outliers (%)	0.43	0.00	0.00	0.00

Statistics for the highest-resolution shell are shown in parentheses. RMSD: root mean square deviation.

Table S1. Crystallographic data collection and refinement statistics (continued).

	Ce149/ BA.2-type RBD (PDB 8YDQ)	CeSPIACE/ BA.2-type RBD (PDB 8YDU)	CeSPIACE/ BA.5-type RBD (PDB 8YDV)	CeSPIACE/ XBB.1.5-type RBD (PDB 8YDW)
**Data collection**				
Space group	$P3_121$	$P3_121$	$P4_1$	$P3_121$
Cell dimensions				
a, b, c (Å)	74.9, 74.9, 100.6	72.3, 72.3, 99.8	87.5, 87.5, 118.2	74.8, 74.8, 100.1
α, β, γ (˚)	90, 90, 120	90, 90, 120	90, 90, 90	90, 90, 120
Resolution (Å)	1.56 (1.65-1.56)	1.63 (1.73-1.63)	2.20 (2.33-2.20)	1.72 (1.82-1.72)
No. of reflections	46951 (7145)	40065 (6419)	45117 (7081)	34940 (5379)
R_{sym}	0.063 (3.568)	0.069 (3.579)	0.157 (2.005)	0.067 (11.980)
CC1/2	99.9 (52.4)	99.9 (56.1)	99.8 (56.4)	99.9 (70.2)
$I/\sigma I$	10.97 (0.40)	10.44 (0.86)	11.99 (1.11)	16.09 (0.32)
Completeness (%)	99.9 (99.7)	99.3 (98.5)	100.0 (100.0)	99.9 (99.9)
Redundancy	6.2 (6.0)	8.3 (8.3)	10.5 (10.6)	13.0 (12.8)
**Refinement**				
Resolution (Å)	39.7-1.9	39.4-1.7	49.0-2.2	39.6-2.0
No. of reflections	26065	35358	45072	22387
R_{work}/ R_{free} (%)	22.7/24.9	22.0/25.2	19.5/22.1	22.3/25.4
No. of atoms	2014	2025	4088	1969
Average B-factor ($Å^2$)	46.0	43.0	51.0	68.0
RMSD				
Bond lengths (Å)	0.00	0.01	0.00	0.01
Bond angles (˚)	0.53	0.71	0.51	1.43
Ramachandran				
Favored (%)	96.54	96.51	98.26	94.83
Allowed (%)	3.46	3.49	1.74	5.17
Outliers (%)	0.00	0.00	0.00	0.00

Statistics for the highest-resolution shell are shown in parentheses. RMSD: root mean square deviation.

Table S2. Roles of CeSPIACE residues. Interaction partner residues are shown in the table. RBD residues were shown basically for the BA.2 strain.

Residues		Peptide stabilization		RBD binding			
		Intramolecular helix bundle	Dimerization	Main chain	Critical site for ACE2 binding	Mutation site	Others
1	D	L3					
2	K	F35, D39					
3	L			A475, G476	N487		
4	W	K8	F35, R38				
5	I	F35	I5, F35				
6	L	M36			A475, Y489		
7	Q	E11		N487	Y489		
8	K	W4, E11	L31				
9	I	V28, I32	I5				
10	Y	M13, V14			Y489		
11	E	Q7, K8					
12	I		M27				
13	M	Y10, V28, S29, I32				Q493 or R493	L455
14	V	Y10				Q493 or R493	
15	R	E19					
16	L	H21, A24, V28	A24, M27				
17	D						Y449
18	E						
19	E	R15					
20	G				R498		
21	H	L16					
22	G				Y501	N501 or Y501	
23	E			Y501, G502	Y501, G502	Y505 or H505	
24	A	L16	L16, A24				
25	S	M13					
26	L			Y495	Y501	N501 or Y501	
27	M		I12, L16			Y505 or H505	
28	V	I9, M13, L16					
29	S	M13					Y453, L455
30	D						R403
31	L		K8				
32	I	I9, M13					L455, F456
33	Y			G416, N417			D420, Y421
34	E						
35	F	K2, I5	W4, I5				
36	M	L6			Y473		F456
37	K						D420
38	R		W4				
39	D	K2					

Table S3. Cryo-EM data collection and refinement statistics.

	CeSPIACE and Ectodomain BA.2 (PDB 8YDX) (EMD-39184)	CeSPIACE and Ectodomain BA.5 class1 (PDB 8YDY) (EMD-39185)	CeSPIACE and Ectodomain BA.5 class2 (PDB 8YDZ) (EMD-39186)
**Data collection**			
Microscope	JEM-Z320FHC	JEM-Z320FHC	JEM-Z320FHC
Detector	K2 Summit	K2 Summit	K2 Summit
Pixel size (Å)	0.96	0.96	0.96
Defocus range (μm)	-1.5 to -2.5	-1.5 to -2.5	-1.5 to -2.5
Voltage (kV)	300	300	300
Total electron dose (e^{-} $Å^{-2}$)	69.4	69.4	69.4
**Reconstruction**			
Final particle images	111,423	18,473	41,878
Pixel size (Å)	1.44	1.44	1.44
Box size (pixels)	340	340	340
Map resolution (Å) (FSC = 0.143)	4.9	8.0	5.2
Map sharpening B-factor ($Å^2$)	-141.5	-114.0	-120.0
**Refinement**			
Model composition			
Non-hydrogen atoms	25,471	24,958	25,303
Protein residues	3,200	3173	3,188
Ligands	NAG: 27	NAG: 4	NAG: 21
Model-to-map CC (mask)	0.63	0.52	0.61
Model-to-map CC (volume)	0.66	0.51	0.63
RMSD			
Bond lengths (Å)	0.011	0.011	0.012
Bond angles (˚)	1.441	1.444	1.441
MolProbity score	1.63	1.71	1.74
All-atom clashscore	8.72	10.85	9.61
Ramachandran plot			
Favored (%)	98.0	97.9	97.7
Allowed (%)	2.0	2.1	2.3
Outliers (%)	0.0	0.0	0.0

CC: correlation coefficient; FSC: Fourier shell correlation; RMSD: root mean square deviation
